# Heterogeneity of *Trypanosoma cruzi* infection rates in vectors and animal reservoirs in Colombia: a systematic review and meta-analysis

**DOI:** 10.1186/s13071-019-3541-5

**Published:** 2019-06-20

**Authors:** Eliana Rodríguez-Monguí, Omar Cantillo-Barraza, Franklin Edwin Prieto-Alvarado, Zulma M. Cucunubá

**Affiliations:** 1 Facultad de Medicina, Universidad El Bosque, Bogotá, Colombia; 2 Grupo de Investigación en Enfermedades Tropicales del Ejercito (GINETEJ), Bogotá, Colombia; 3 Department of Infectious Disease Epidemiology, MRC Centre for Global Infectious Disease Analysis (MRC GIDA), Imperial College London, London, UK

**Keywords:** Chagas disease, *Trypanosoma cruzi*, Triatomines, Reservoirs, Infection rates, Heterogeneity

## Abstract

**Background:**

The heterogeneity of *Trypanosoma cruzi* infection rates among triatomines insects and animal reservoirs has been studied in independent studies, but little information has been systematised to allow pooled and comparative estimates. Unravelling the main patterns of this heterogeneity could contribute to a further understanding of *T. cruzi* transmission in Colombia.

**Methods:**

A systematic search was conducted in PubMed, Medline, LILACS, Embase, Web of Knowledge, Google Scholar and secondary sources with no filters of language or time and until April 2018. Based on selection criteria, all relevant studies reporting *T. cruzi* infection rates in reservoirs or triatomines were chosen. For pooled analyses, a random effects model for binomial distribution was used. Heterogeneity among studies is reported as *I*^2^. Subgroup analyses included: taxonomic classification, ecotope and diagnostic methods. Publication bias and sensitivity analyses were performed.

**Results:**

Overall, 39 studies reporting infection rates in Colombia were found (22 for potential reservoirs and 28 for triatomine insects) for a total sample of 22,838 potential animals and 11,307 triatomines evaluated for *T. cruzi* infection. We have found evidence of 38/71 different animal species as potential *T. cruzi* reservoirs and 14/18 species as triatomine vectors for *T. cruzi*. Among animals, the species with the highest pooled prevalence were opossum (*Didelphis marsupialis*) with 48.0% (95% CI: 26–71%; *I*^2^ = 88%, *τ*^2^ = 0.07, *P* < 0.01) and domestic dog (*Canis lupus familiaris*) with 22.0% (95% CI: 4–48%; *I*^2^ = 96%, *τ*^2^ = 0.01, *P* < 0.01). Among triatomines, the highest prevalence was found for *Triatoma maculata* in the peridomestic ecotope (68.0%, 95% CI: 62–74%; *I*^2^ = 0%, *τ*^2^ = 0, *P* < 0.0001), followed by *Rhodnius prolixus* (62.0%, 95% CI: 38–84%; *I*^2^ = 95%, *τ*^2^ = 0.05, *P* < 0.01) and *Rhodnius pallescens* (54.0%, 95% CI: 37–71%; *I*^2^ = 86%, *τ*^2^ = 0.035, *P* < 0.01) in the sylvatic ecotope.

**Conclusions:**

To our knowledge, this is the first systematic and quantitative analyses of triatomine insects and potential animal reservoirs for *T. cruzi* infection in Colombia. The results highlight a marked heterogeneity between species and provide initial estimates of infection rates heterogeneity.

**Electronic supplementary material:**

The online version of this article (10.1186/s13071-019-3541-5) contains supplementary material, which is available to authorized users.

## Background

Chagas disease is caused by the protozoan *Trypanosoma cruzi*, a parasite largely distributed across 21 Latin American countries where it has been associated with increased morbidity and mortality and a high economic burden [1, 2]. Transmission through triatomine vectors is the most frequent route, followed in frequency by congenital, blood transfusions, transplants and oral mechanisms [3].

*Trypanosoma cruzi* transmission is highly complex as it is the cause of a zoonotic parasitosis present in more than 150 animal reservoirs and 147 triatomine vectors [4, 5]. Additionally, six different discreet typing units (DTUs) of *T. cruzi* have been described so far [4, 6, 7]. The role that different animals and triatomine species play in *T. cruzi* transmission to humans depends on the local conditions that facilitate contact with humans in the different type of cycles: sylvatic, peridomestic and domestic [8]. Importantly, domestic transmission from non-native triatomines is subject to control *via* insecticide spraying and housing improvement, and there has been a great success controlling these vectors across Latin American countries [9–11]. However, sylvatic transmission is much more complex and control measures are less clear [12]; continued population growth and deforestation across sylvatic areas increase the likelihood that humans establish closer contact with both animal reservoirs and triatomine vectors from the sylvatic cycles [8]. Furthermore, there is an increased report of outbreaks arising from oral transmission in both rural areas and major urban populations of Brazil, Colombia and Venezuela, also suggesting an increased contact between sylvatic and domestic ecotopes [13].

Among sylvatic mammals, the most frequently reported species associated to *T. cruzi* transmission are from the order Didelphimorphia, and particularly the species *Didelphis marsupialis* [14–17]. These animals are considered transmission amplifiers due to their generally high parasitaemia and easy adaptation to disturbed areas [18]. Other sylvatic orders with species reported as hosts include Cingulata (*Dasypus novencintus*), Rodentia (*Akodon* spp., *Dasyprocta* spp.), Chiroptera (*Carollia perspicillata*, *Artibeus fuliginosus*) and primates (*Ateles* spp., *Cebus* spp.) [7]. Among domestic mammals, the species of the orders Carnivora (*Canis lupus familiaris*, *Felis silvestris catus*) and Rodentia (*Rattus rattus*, *Rattus norvegicus* and *Cavia* spp.) have been reported with high infection rates, whereas large mammals from the orders Artiodactyla (subfamilies Bovinae, Caprinae and Suinae) and Perissodactyla (family Equidae) are rarely infected or show very low infection rates in South America [7].

In Colombia, 26 triatomine species have been reported as potential vectors for *T. cruzi*. Among them, *Rhodnius prolixus*, *Triatoma dimidiata*, *T. maculata* and *T. venosa* are the most frequently found close to humans, within houses or in the surrounding area (peridomicile) [6, 19]. However, a large number of sylvatic species have the capacity to occasionally invade homes and to lead to human infection. The importance of these species is indicated by their distribution, density, transmission efficiency, feeding behaviour and the prevalence of infection [20, 21]. The main triatomine species in domiciliated habitats in Colombia is *R. prolixus.* Important efforts to interrupt domestic transmission from this species to humans have been made in the last decades and great progress has been made in some parts of the country [22]. However, Chagas outbreaks resulting from oral transmission have been continuously reported in recent decades, highlighting the importance of gaining a better understanding of the sylvatic cycle [16].

Documenting and quantifying general trends and heterogeneity in infection rates among the different reservoir hosts and triatomine vector species is paramount to understanding the role that these species play in *T. cruzi* transmission to humans. Here, we conducted a literature review and meta-analysis of the existent evidence for *T. cruzi* presence and infection rates in vectors and animal reservoirs in Colombia.

## Methods

The systematic review and meta-analysis were carried out following the verification checklist PRISMA to ensure standardised methods (Additional file 1).

### Search strategy

A search for observational studies of *T. cruzi* infection among animal reservoirs and triatomine vectors was conducted in PubMed, Medline, Index Medicus, LILACS, Embase and Web of Knowledge without language or time filter until 5th April 2018. Additionally, a secondary search through Google Scholar and Colombia University thesis repositories was conducted. For details of the search algorithm, see Additional file 2: Table S1.

### Selection criteria and data extraction

Selection criteria included: (i) study type as cross-sectional studies that included triatomines or potential animal reservoirs; (ii) conduced in Colombian territory; (iii) reporting details of the number of tested individuals and the number of positive individuals; (iv) with details on diagnostic method, ecotope of capture; (v) with a minimum sample size of 10 subjects for pooled calculations.

An Excel spreadsheet was used to report characteristics of the studies, including reference, year of the study, place, ecotope, sample size, diagnostic method and taxonomic classification. For quantitative analyses, both triatomines and potential animal reservoirs were grouped by taxonomic characteristics (order, family, genus and species) as much as possible.

Diagnostic methods for potential reservoirs were classified as: (i) serological methods that do not account for current infection and have high cross-reactivity with other tripanosomatid species; (ii) non-serological methods, including both parasitological (blood spread smear, hemoculture and xenodiagnostic) and molecular methods (PCR), that allow establishing solid evidence as *T. cruzi* reservoirs; and (iii) mixed methods, which use a combination of the two previous methods but with no clear information to allow disaggregated data.

Diagnostic methods for triatomine vectors were classified as: (i) *T. cruzi* species-specific (PCR, culture and mice inoculation); and (ii) *T. cruzi* non-species-specific (i.e. microscopic examination of extruded faeces), as *T. rangeli* or other flagellates such as *Crithidia* could confound diagnostic [23–25].

### Quality assessment

A content quality assessment was conducted. The items evaluated included research question, sampling method, study period, diagnostic method, and potential bias. We allocated a score to each item in order to create an overall classification of high, moderate, or low quality [26] (see Additional file 2: Figure S1).

### Statistical analysis

#### Descriptive statistics

Descriptive statistics are presented as proportion of infection and 95% confidence intervals (95% CI). Frequency tables and maps of geographical distribution are presented.

#### Pooled analysis

A meta-analysis of binary outcome with binomial distribution was conducted in R v.3.2.2. specifically using the ‘meta’ library [27]. In order to stabilise variance a Freeman-Tukey double-arccosine transformation was used [27, 28]. Given the fact that the study design used for the multiple species, the sample size, geographical characteristics and methods used could differ between studies, a random effects model was considered more appropriate [29], where tau-squared (*τ*^2^) represents the variance between studies [27, 29]. For assessing heterogeneity between studies, the *I*^2^- and Cochranʼs Q tests are presented. *I*^2^ indicates the proportion of the total variation attributed to heterogeneity, i.e. an assessment of the degree of coherence between studies [*I*^2^ = 100% × (Q − df)/Q]. The Cochranʼs Q is calculated as the sum of the squared deviations of each study’s estimate that allows weighting each study’s contribution [30].

#### Publication bias and sensitivity analysis

Publication bias was assessed when possible (for meta-analysis with at least five studies included), by using the Eggerʼs test, funnel plots and Trim fill technique evaluating the potential effect of non-included studies on the general estimates [31]. Sensitivity analysis was conducted by re-assessing general effect while sequentially omitting one study at a time [32] and also restricting the analyses to only high quality studies.

## Results

### General findings

**Fig. 1:**
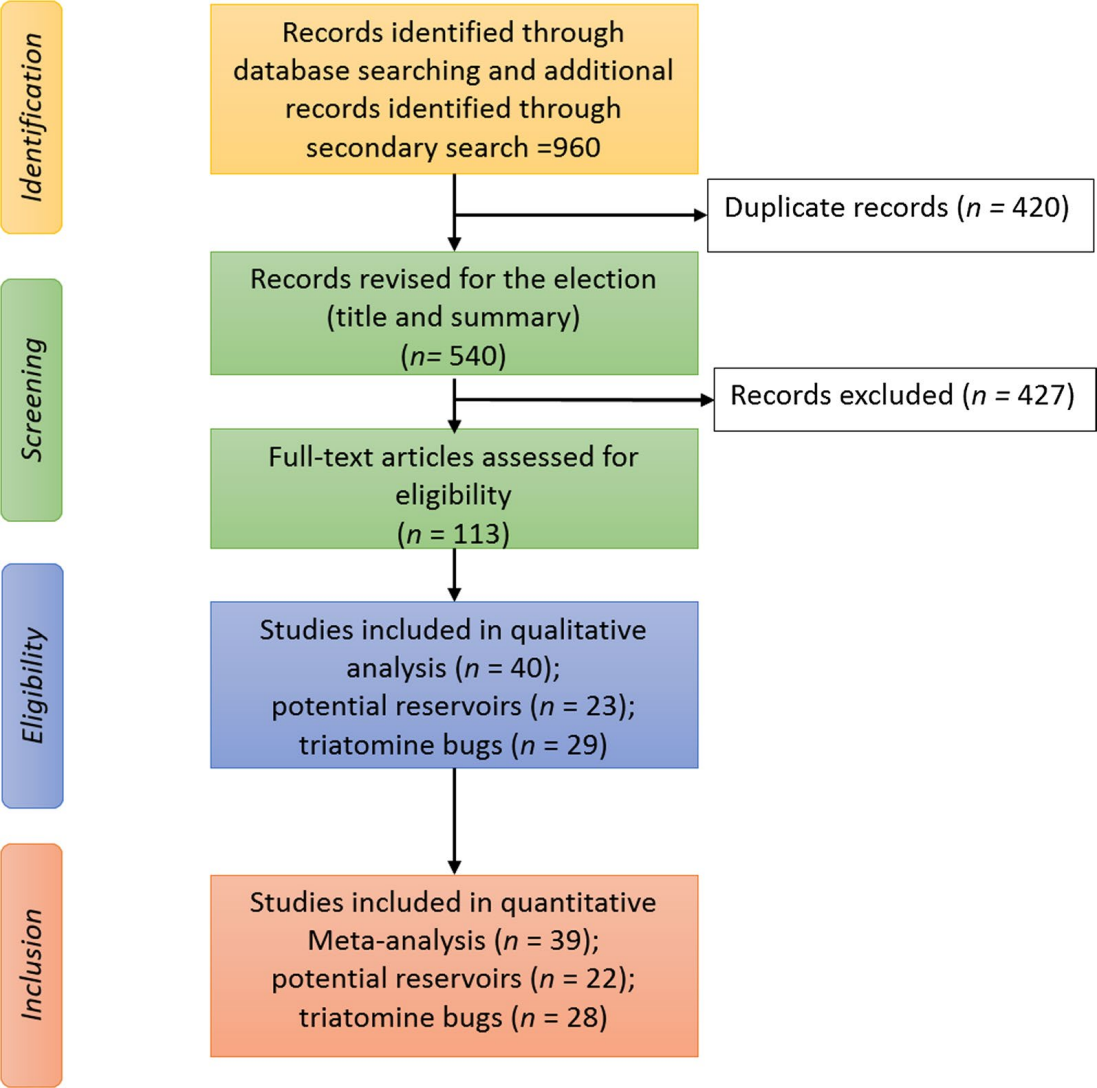
Flow diagram describing the search and selection process for both qualitative and quantitative analysis

Overall, 960 potentially useful studies were found. After reading all abstracts and based on the selection criteria indicated above, 40 studies containing relevant information of the presence of *T. cruzi* infection in animals or triatomines were selected, including one study on culture parasite isolates [33] that was relevant for qualitative analyses but not for prevalence calculations. Therefore, for prevalence calculations we used 39 studies; of these, 22 included data for prevalence in potential animal reservoir and 28 for prevalence in triatomine vectors (see Fig. 1 for details). Table 1 shows a general description of the final 39 studies included in the quantitative analyses.

**Table 1:** General information on the 39 studies included for quantitative analysis of *T. cruzi* infection in potential animal reservoirs and triatomine vectors

References	Study period	Department	No. mun (*n*)	Triatomine bugs (*n*)	Potential reservoirs (*n*)
Wolff et al. 2001 [79]	1997–1999	Antioquia	8	124	70
Escalante et al. 2015 [80]	2013	Atlántico, Bolívar, Córdoba, Sucre	4	89	0
Parra-Henao et al. 2015 [81]	2002–2014	Atlántico, Boyacá, Caquetá, Guajira	77	4943	0
Cortéz et al. 2005 [82]	2003	Bolívar	1	30	0
Cantillo-Barraza et al. 2010 [63]	2006–2008	Bolívar	6	43	0
Cantillo-Barraza et al. 2015 [61]	2010–2012	Bolívar	5	113	267
Vásquez et al. 2013 [77]	2003–2004	Bolívar, Magdalena	6	66	115
Manrique et al. 2012 [83]	2009	Boyacá	2	0	60
Ramírez et al. 2013 [84]	2012	Boyacá	2	0	80
Ramírez et al. 2014 [85]	2010	Casanare	6	0	175
Rendón et al. 2015 [51]	2012	Casanare	2	269	177
Urbano et al. 2015 [59]	2012	Casanare	1	169	0
Jácome-Pinilla et al. 2015 [65]	2012	Casanare	1	26	0
Angulo-Silva et al. 2016 [46]	2008	Casanare	2	56	35
Zuleta-Dueñas et al. 2017 [38]	2014	Casanare	1	0	4
Angulo et al. 2012 [58]	2003–2006	Casanare, Arauca	4	113	0
DʼAlessandro 1971 [86]	1968	Casanare, Vichada, Meta	3	0	11
Sandoval et al. 2004 [87]	2001	Cesar	2	44	0
Montilla et al. 2011 [88]	2006–2007	Cesar	19	3107	0
Soto et al. 2014 [37]	2010	Cesar	1	135	2
Parra et al. 2004 [89]	1999	Cesar, Guajira, Magdalena	2 (V) SNSM	70	1
Parra-Henao et al. 2009 [90]	2006–2008	Cesar, Guajira, Magdalena	8 (V)	207	0
Mejía-Jaramillo et al. 2014 [91]	2011	Cesar, Guajira, Magdalena	4 (V) SNSM	463	151
Parra-Henao et al. 2016 [92]	2007–2008	Cesar, Guajira, Magdalena	18	225	0
Marinkelle 1982(a) [93]	1961–1972	Colombia	NM	0	343
Marinkelle 1982(b) [50]	1961–1972	Colombia	658 (L)	0	19885
Delgado-Sarmiento et al. 2016 [94]	2013	Córdoba	4 (L)	0	30
Peña-García et al. 2014 [95]	2010–2011	Guajira	1	78	12
Gómez-Melendro et al. 2014 [60]	2013	Guajira	2	32	0
Cantillo-Barraza et al. 2014 [66]	2011	Isla Margarita	5	0	27
Dib 2009 [96]	2006	Magdalena	1	35	0
Salazar et al. 2003 [97]	2003	Magdalena	7 (V)	73	0
D’Alessandro et al. 1984 [71]	1978–1979	Meta	2	384	756
Jaimes-Dueñez et al. 2017 [78]	2015	Meta	1	0	242
Hernández et al. 2016 [5]	2013	Meta, Guajira, Cesar, Antioquia, Norte de Santander, Casanare, Huila	7	245	0
Travi et al. 1994 [98]	1991	Nariño, Córdoba	2	0	394
Sandoval et al. 2010 [70]	2004–2006	Norte de Santander	1	123	0
Reyes et al. 2017 [15]	2014	Santander	1	17	1
Castro-Salas et al. 2013 [64]	2010	Vichada	1	28	0

*Notes*: Letters (a) and (b) refer to different studies by the same first author in a single year. The study by León et al. [33] was included as part of the qualitative analyses (Tables 2 and 3) but not as part of the meta-analyses as this is a study on parasite isolates only

*Abbreviations*: mun, municipalities; SNSM, Sierra Nevada de Santa Marta; L, localities; V, villages

**Fig. 2:**
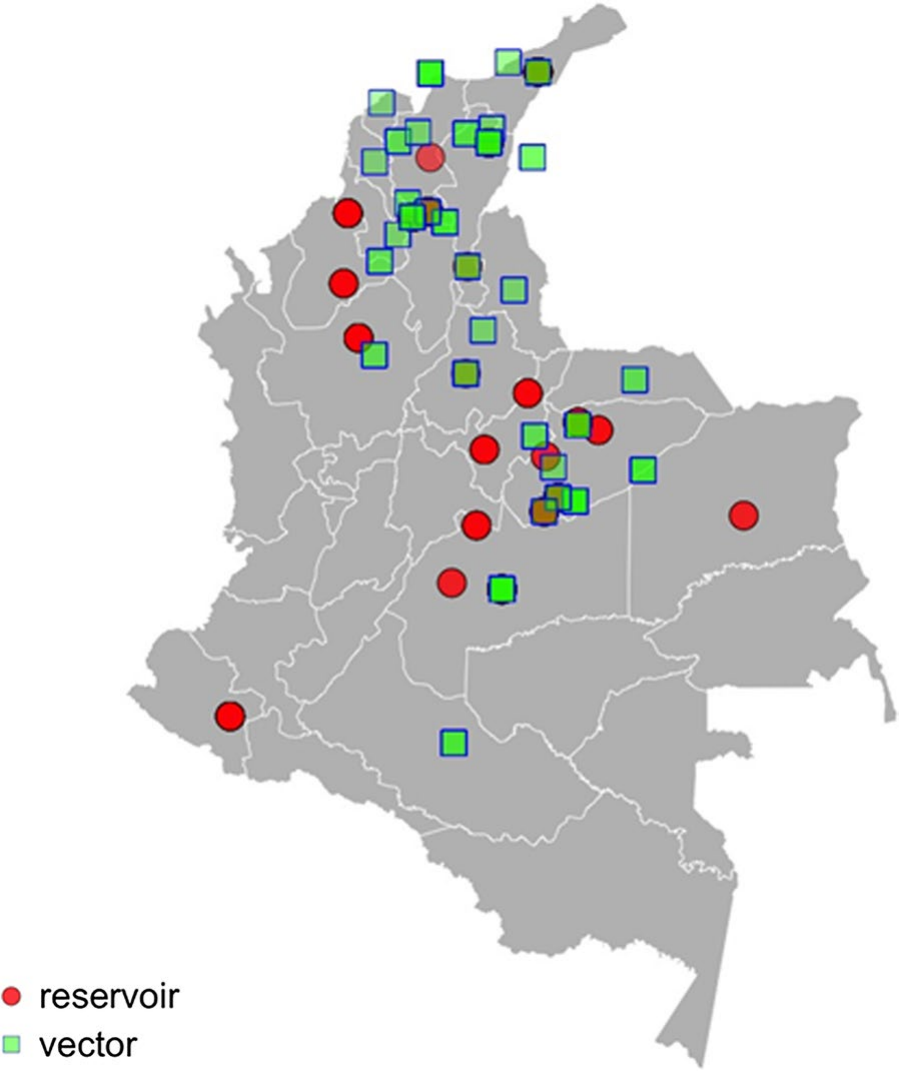
Map showing the geographical distribution of the samples used in individual studies for both animal reservoirs (red circles) and triatomine vectors (green pictures) investigated for *T. cruzi* infection in Colombia

Geographical distribution of the samples used in the studies for potential animal reservoirs and triatomine vectors include 9 and 16 departments (first administrative level), respectively, and also the special territory of the Sierra Nevada de Santa Marta (SNSM). The departments with the highest number of studies were Casanare, Córdoba, Meta and Bolívar for reservoirs and Casanare, Bolívar, César, Guajira, Magdalena and SNSM for triatomines (see Fig. 2) (for details, see Additional file 2: Table S2.

### Potential reservoirs

**Fig. 3:**
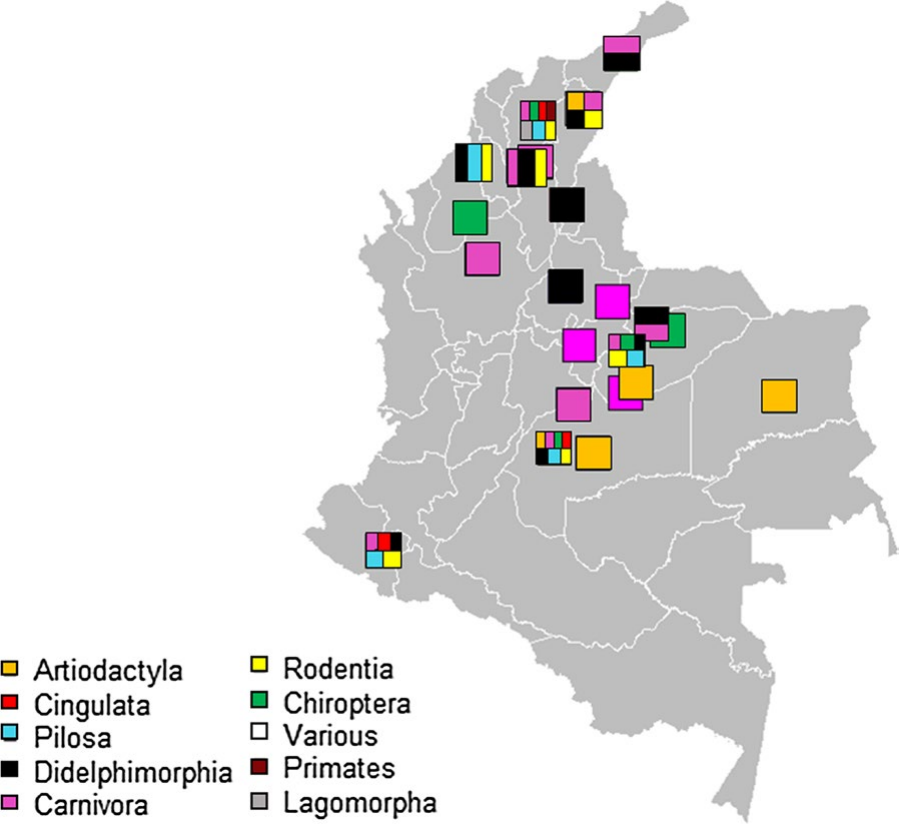
Geographical distribution of studies of potential animal reservoirs of *T. cruzi* infection by taxonomic order

The number of subjects (potential animal reservoirs) analysed in the 22 selected studies was 22,838 of which 1585 (6.94%) were found infected with *Trypanosoma* spp. Eighteen of these studies reported the capture method; Sherman^®^ and Tomahawak^®^ were the most commonly used. All 22 studies reported the ecotope of collection and diagnostic method. The geographical distribution of the studies included classified by taxonomic order are shown in Fig. 3. Overall, 71 animal species of nine mammal orders were studied, out of which 38 were found infected with *T. cruzi* (Table 2).

**Table 2:** Taxonomic classification of potential animal reservoir and evidence of infection with *T. cruzi* in Colombia

Family	Genus	Species	Common name (as used in Colombia)	*T. cruzi* infection
*Order Artiodactyla*				
Cervidae	*Mazama*	*M. gouazoubira medemi*	Viracho	Yes
	*Odocoileus*	*O. virginianus*	Venado de cola blanca	Yes
Suidae	*Sus*	*S. scrofa*	Jabalí	No
Tayassuidae	*Tayassu*	*T. albirostris*	Pecarí barbiblanco o baquira	No
*Order Carnivora*				
Canidae	*Canis*	*C. lupus*	Perro	Yes
	*Cerdocyon*	*C. thous*	Zorro cangrejero	No
Felidae	*Felis*	*F. concolor*	Puma	No
		*F. pardalis*	Ocelote	No
		*F. silvestris catus*	Gato doméstico	Yes
Procyonidae	*Potos*	*P. flavus*	Perro de monte	Yes
*Order Chiroptera*				
Phyllostomidae	*Artibeus*	*A. fuliginosus*	Murciélago frugívoro neotropical	Yes
		*A. jamaicensis*	Murciélago frugívoro de Jamaica	Yes
		*A. lituratus*	Murciélago frugívoro neotropical	Yes
		*A. planirostris*	Murciélago frugívoro neotropical	Yes
		*A. phaeotis*	Murciélago frugívoro neotropical	Yes
	*Carollia*	*C. perspicillata*	Murciélago común frugívoro	Yes
	*Desmodus*	*D. rotundus*	Vampiro de Araza	Yes
	*Glossophaga*	*Glossophaga* sp.	Murciélago neotropicales	Yes
	*Phyllostoma*	*P. discolour*	Murciélago nariz de lanza	No
	*Sturnira*	*Sturnira.* sp.	Murciélago microquiróptero	No
Vespertilionidae	*Eptesicus*	*E. furinalis*	Murciélago pardo común	Yes
	*Lasiurus*	*L. borealis*	Murciélago Colorado	No
	*Myotis*	*M. nigricans*	Murciélago negro pequeño	No
	*Myotis*	*M. oxyotus*	Murciélago negro pequeño	Yes
Molossidae	*Eumops*	*E. perotis*	Murciélago gigante de bonete	No
	*Molossus*	*M. bondae*	Murciélago mastín de Bonda	Yes
		*M. major*	Murciélago de cola libre insectívoro	No
		*M. molossus*	Murciélago mastín común	No
Noctilionidae	*Noctilio*	*N. labialis*	Murciélago Pescadores	No
		*N. leporinus*	Murciélago pescador	No
Emballonuridae	*Rhynchonycteris*	*R. naso*	Murciélago narigón	Yes
*Order Cingulata*				
Dasypodidae	*Dasypus*	*D. kappleri*	Armadillo	Yes
		*D. novemcinctus*	Armadillo de 9 bandas	Yes
		*D. sabanicola*	Cachicamo Sabanero	Yes
	*Proechymis*	*P. giganteus*	Armadillo gigante	No
*Order Lagomorpha*				
Leporidae	*Oryctolagus*	*O. cuniculus*	Conejo	Yes
*Order Didelphimorphia*				
Didelphidae	*Didelphis*	*D. marsupialis*	Zarigueya común	Yes
	*Lutrolina*	*L. crassicaudata*	Zarigueya de cola gruesa	No
	*Marmosa*	*M. andersoni*	Otras zarigüeyas	No
		*M.* cf. *robinsoni*	Marmosa de Robinson	Yes
		*M. murina*	Zarigueya ratón	No
	*Metachirus*	*M. nudicaudatus*	Comadreja de anteojos	Yes
	*Monodelphis*	*Monodelphis* sp.	Zarigueyas de pequeños tamaños	No
	*Philander*	*P. opossum*	Zarigueya gris de cuatro ojos	No
	*Caluromys*	*C. lanatus*	Zarigüeya lanuda oocidental	Yes
*Order Pilosa*				
Bradypodidae	*Bradypus*	*B. variegatus*	Perezoso bayo	No
Megalonychidae	*Choloepus*	*C. hoffmanni*	Perezoso de dos dedos de Hoffmann	No
Cyclopedidae	*Cyclopes*	*C. didactylus*	Oso hormiguero pigmeo	No
Myrmecophagidae	*Myrmecophaga*	*M. tridactyla*	Oso hormiguero gigante	Yes
	*Tamandua*	*T. tetradactyla*	Oso hormiguero de chaleco	Yes
		*T.mexicana*	Oso hormiguero	Yes
*Order Primates*				
Atelidae	*Alouatta*	*A. caraya*	Primates	No
*Order Rodentia*				
Caviidae	*Cavia*	*C. porcellus*	Cuy	No
Erethizontidae	*Coendou*	*Coendou* sp.	Puercoespin americano de cola larga	No
Cuniculidae	*Cuniculus*	*C. paca*	Paca común, lapa	Yes
Dasyroctidae	*Dasyprocta*	*D. fuliginosa*	Ñeque o picure	Yes
		*D. punctata*	Ñeque o guatín	No
Heteromyidae	*Heteromys*	*H. anomalus*	Ratón mochilero	Yes
Echimyidae	*Hoplomys*	*H. gimnurus*	Rata acorazada	No
Caviidae	*Hydrochoerus*	*H. hydrochaeris*	Chiguiro o Capibara	Yes
Muridae	*Mus*	*M. musculus*	Ratón casero	No
Cricetidae	*Neacomys*	*N. spinosus*	Roedor miomorfo	No
	*Nectomys*	*N. squamipes*	Rata de agua brasileña	No
	*Zygodontomys*	*Z. brevicauda*	Ratones de las cañas, pichunas	No
		*Z. brunneus*	Ratones de las cañas, pichunas	Yes
	*Sigmodon*	*S. alstoni*	Rata de algodón	No
	*Tylomys*	*T. mirae*	Rata trepadora Mira	Yes
	*Oryzomys*	*O. concolor*	Ratones de arrozal	Yes
		*O. delicates*	Ratones de arrozal	No
		*O. fulvescens*	Ratones de arrozal	No
Echimyidae	*Proechymis*	*P. semiespinosus*	Ratas espinosas, ratones de monte	Yes
		*P. oconnelli*	Guira, ratas espinosas, ratones de arrozal	No
	*Diplomys*	*Diplomys* sp.	Rata Espinosa	Yes
Muridae	*Rattus*	*R. rattus*	Rata negra o rata común	Yes
		*R. norvegicus*	Rata gris o rata de alcantarilla	No

**Fig. 4:**
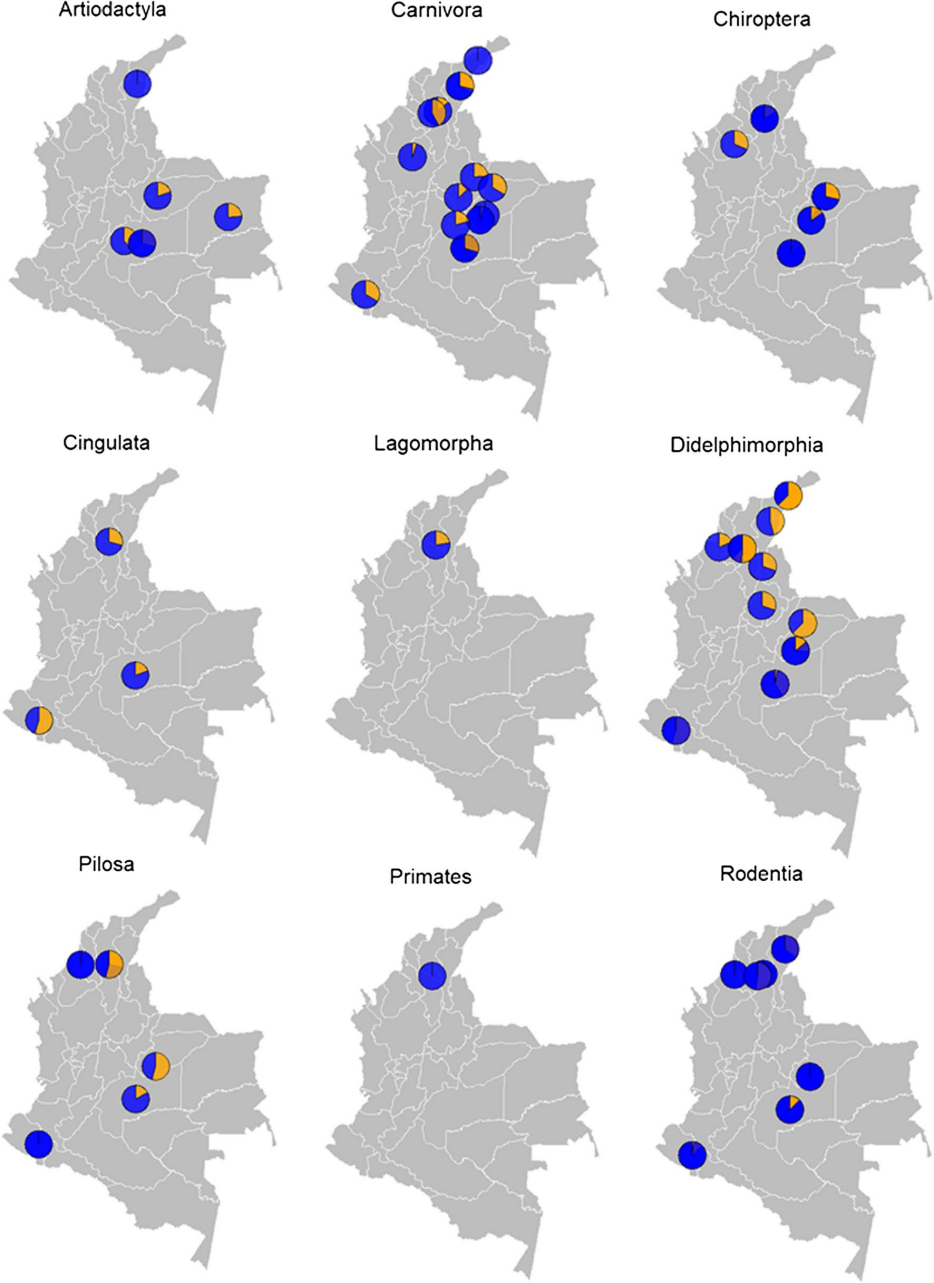
Geographical distribution of most relevant animal reservoirs studied, by taxonomic order. The pies within the maps represent the proportion of *Trypanosoma* spp. infections (orange) and negative samples (blue) from each study

The geographical distribution of potential animal reservoirs and proportion of infection found by location by taxonomic order are presented in Fig. 4. Further details on the general characteristics of the studies and proportion of infection are shown in Additional file 2: Table S3.

### Triatomine insects

The total sample size among studies included was 11,307 triatomines of which 1721 (15.22%) presented *Trypanosoma* spp. infection; of these, 1354 (11.97%) presented infection by *T. cruzi.* The capture protocols most commonly used were active search and live bait traps. Out of the 28 studies, 23 registered disaggregated data for ecotope of collection. Diagnostic methods used were mainly parasitological (direct microscopic examination of extruded faeces) followed by PCR and mice inoculation (see Additional file 2: Table S4) for further details.

**Fig. 5:**
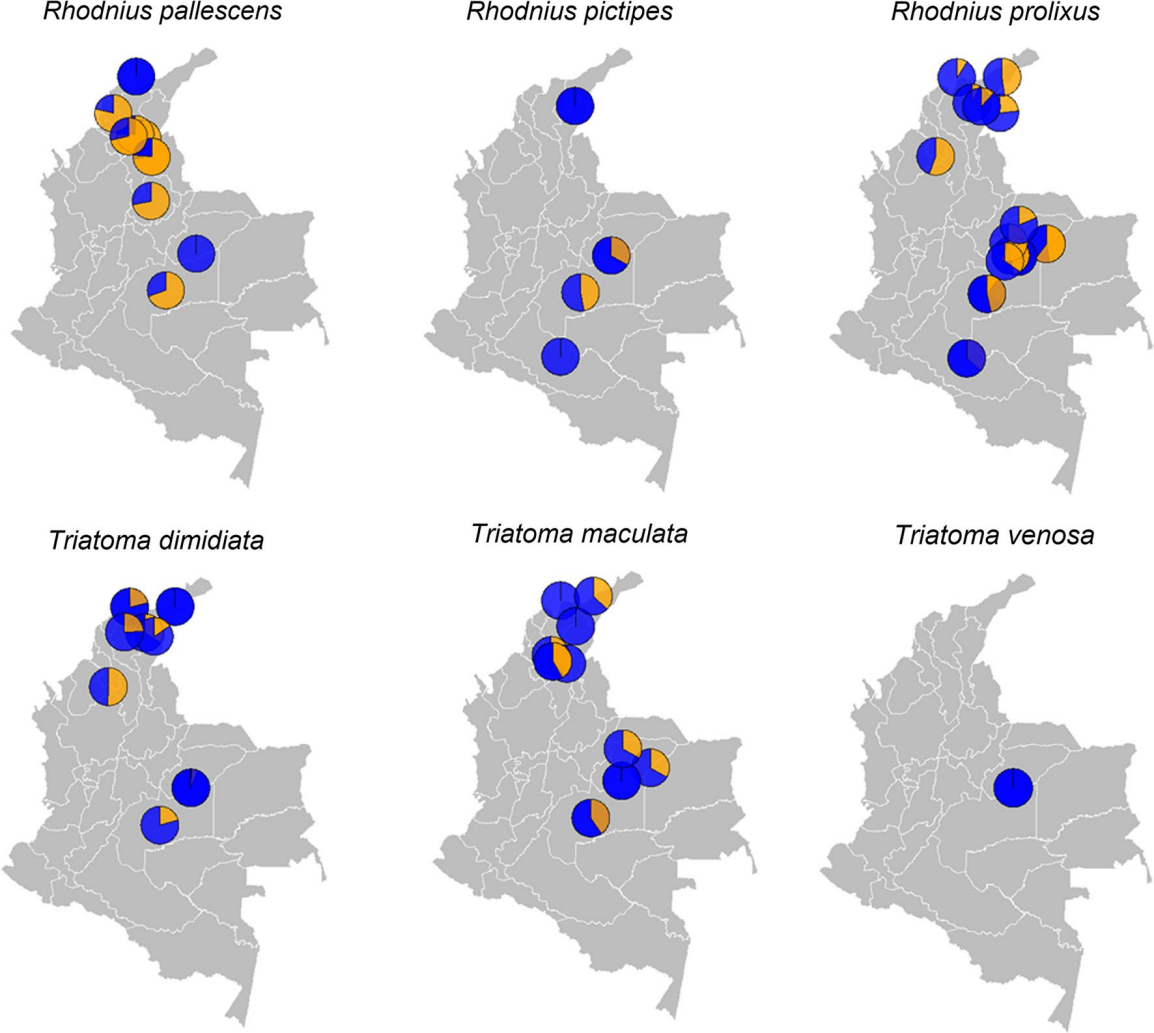
Geographical distribution of the six most relevant triatomine species studied. The pies within the maps represent the proportion of *Trypanosoma* spp. infections (orange) and negative samples (blue) from each study

In the selected papers, 18 triatomine species were studied of which four (*Belminus ferroae*, *Eratyrus mucronatus*, *Psammolestes arthuri* and *Panstrongylus lignarius*) were found with no evidence and 14 with solid evidence of *T. cruzi* infection (Table 3). Table 3 shows the geographical distribution of all triatomine species studied, except for *Triatoma dispar*, *Rhodnius robustus* and *R. colombiensis* as the studies reporting these species did not have geographical information. The point prevalence of *Trypanosoma* spp. in triatomine insects and the geographical distribution is presented in Fig. 5.

**Table 3:** Evidence of triatomine species studied for *T. cruzi* infection in Colombia

Genus	Species	Evidence of *T. cruzi* infection	Geographical distribution
*Belminus*	*B. ferroae*	No	Norte de Santander
	*B. herreri*	Yes	Cesar
*Eratyrus*	*E. mucronatus*	No	Boyaca
	*E. cuspidatus*	Yes	Atlantico, Bolivar, Magdalena
*Cavernicola*	*C. pilosa*	Yes	Meta
*Psammolestes*	*P. arthuri*	No	Casanare, Meta
*Panstrongylus*	*P. lignarius*	No^a^	Meta
	*P. geniculatus*	Yes	Atlantico, Boyaca, Casanare, Magdalena, Meta, Santander, SNSM, Sucre, Vichada
	*P. rufotuberculatus*	Yes	Antioquia, Boyaca, SNSM
*Rhodnius*	*R. pallescens*	Yes	Bolivar, Boyaca, Cesar, Magdalena, Santander, SNSM
	*R. prolixus*	Yes	Boyaca, Caqueta, Casanare, Cordoba, Guajira, Meta, SNSM, Vichada
	*R. pictipes*	Yes	Boyaca, Caqueta, SNSM
	*R. robustus*	Yes^b^	
	*R. colombiensis*	Yes^b^	
*Triatoma*	*T. dimidiata*	Yes	Boyaca, Cordoba, Guajira, SNSM
	*T. maculata*	Yes	Bolivar, Boyaca, Casanare, Guajira, Magdalena, Meta, SNSM, Vichada
	*T. venosa*	Yes	Boyaca
	*T. dispar*	Yes^b^	

^a^There is evidence of infection with *Trypanosoma* spp., but not confirmed evidence of *T. cruzi* due to the diagnostic method used (microscopic examination of extruded faeces)

^b^Obtained from studies on isolates of *T. cruzi* parasites

*Abbreviations*: SNSM, Sierra Nevada de Santa Marta

### Pooled analyses

#### Pooled prevalence of *T. cruzi* infection in animal reservoirs

Out of the nine orders of potential reservoirs reported in this study, seven presented sufficient sample size and additional information for calculating pooled estimates of the prevalence of *T. cruzi* infection. The aggregated estimates showed that the species with highest prevalence was *Didelphis marsupialis*, followed by *Canis familiaris* (Table 4). Further details of the disaggregated results are shown in Additional file 2: Table S5.

**Table 4:** Results of point or pooled prevalence estimates (meta-analysis using random effects model) by serological *vs* non-serological methods for the different orders of potential *Trypanosoma cruzi* animal reservoirs studied in Colombia

Order/family/species	Diagnostic subgroup	No. of studies	Potential reservoirs (*n*)	Potential reservoirs (+)	Pooled prevalence (%)	95% CI	*I*^2^ (%)	*τ* ^2^	*P*-value
Carnivora	Non-serological	11	940	347	17	5–33	96	0.08	<0.01
Family Canidae/*Canis lupus familiaris*	Serological	4	430	196	24	1–64	98	0.17	<0.01
	Non-serological	5	257	89	14	1–37	93	0.07	<0.01
	Mixed	1	242	62	26	2–31	–	–	–
Family Felidae	Non-serological	1	11	0	0	0–15	–	–	–
Chiroptera	Non-serological	6	20,267	1010	15	5–29	96	0.04	<0.01
Cingulata	Non-serological	1	21	4	19	5–39	–	–	–
Pilosa	Non-serological	1	46	0	0	0–4	–	–	–
Didelphimorphia	Non-serological	5	310	80	35	16–57	92	0.06	<0.01
*Didelphis marsupialis*	Non-serological	5	161	73	48	26–71	88	0.07	<0.01
Other *Didelphis*	Non-serological	3	148	6	3	0–10	62	0.09	0.07
Primates	Non-serological	1	343	53	15	12–19	–	–	–
Rodentia	Non-serological	5	799	59	6	2–12	77	0.01	<0.01
Other non-classified species	Non-serological	1	10	2	20	1–51	–	–	–

*Notes*: Non-serological: includes parasitological methods (blood smears hemoculture and xenodiagnostic) and molecular methods (PCR)

Serological: includes ELISA, IFAT or rapid tests based on the whole parasite or recombined antigens; +: positive

Two orders, Artiodactyla and Lagomorpha, were excluded from the quantitative analyses due to small sample size. Within the order Artiodactyla (i.e. white-tailed deer, collared peccary and boar), a prevalence ranging between 0–5% was registered. For the order Lagomorpha, only one study was found for the European rabbit *Oryctolagus cuniculus* with a prevalence of 22% (2/9).

**Fig. 6:**
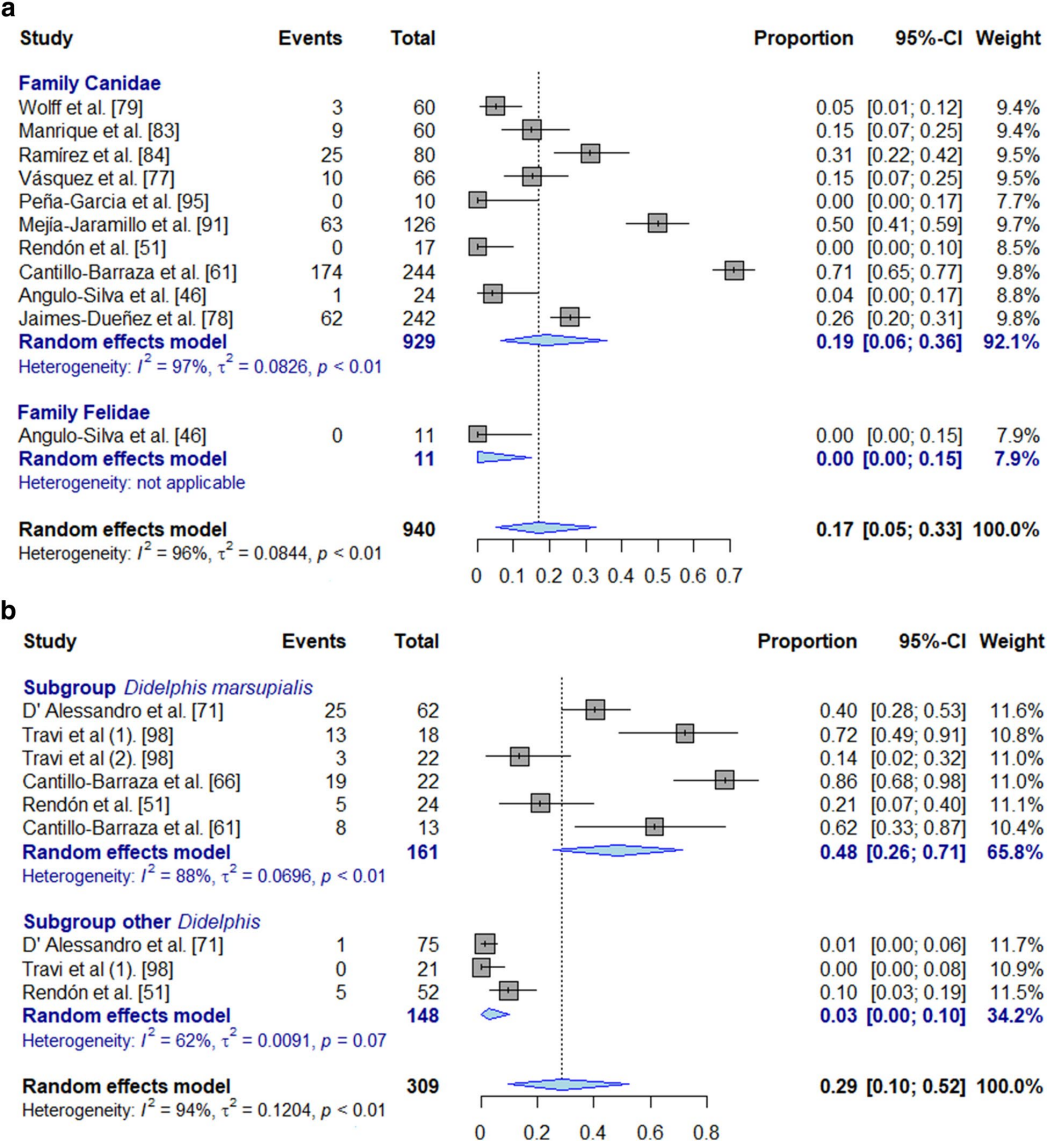
Meta-analysis for pooled prevalence of *T. cruzi* infection using random effects model for the orders Carnivora (**a**) and Didelphimorphia (**b**) without disaggregation by diagnostic tests used. Numbers 1 and 2 in parentheses indicate the same study but a different geographical distribution

The two orders with the highest number of studies were Didelphimorphia and Carnivora, with 11 studies each. The results are presented in Fig. 6. Not all of these studies were included in meta-analyses due to sample size restrictions. Eleven studies reported Didelphimorphia. However, only five of them were included for pooled estimates due to sample size restrictions. The estimated prevalence for common opossum (*Didelphis marsupialis)* was 48.0% (95% CI: 26–71%; *I*^2^ = 88%, *τ*^2^ = 0.07, *P* < 0.01). The order Carnivora was the only one with reports for prevalence based on serological tests. The most relevant species, the domestic dog (*Canis lupus familiaris*), presented a high pooled prevalence of *T. cruzi* infection measured by serological tests (24.0%, 95% CI: 1–64%; *I*^2^ = 98%, *τ*^2^ = 0.17, *P* < 0.01). Only one study included the kinkajou (*Potos flavus*) and presented a point prevalence of *T. cruzi* of 44.4%. The order Chiroptera (bats) and the order Rodentia (rodents) were found with a pooled prevalence of 15.0% (95% CI: 5–29%; *I*^2^ = 96%, *τ*^2^ = 0.04, *P* < 0.01) and 6.0% (95% CI: 2–12%, *I*^2^ = 77%, *τ*^2^ = 0.01, *P* < 0.01), respectively.

#### Pooled prevalence of *T. cruzi* infection in triatomines

Among the 18 triatomine species studied, the highest pooled prevalence for *T. cruzi* infection was found for *T. maculata* 68.0% (95% CI: 62–74%; *I*^2^ = 0.0%, *τ*^2^ = 0.0, *P* < 0.0001), followed by sylvatic *R. prolixus* 62.0% (95% CI: 38–84%; *I*^2^ = 95%, *τ*^2^ = 0.05, *P* < 0.01) and sylvatic *R. pallescens* 54.0% (95% CI: 31–71%; *I*^2^ = 86%, *τ*^2^ = 0.03, *P* < 0.01). Another species of great public health importance, *T. dimidiata*, presented a pooled prevalence of 20.0% (95% CI: 8–35%; *I*^2^ = 90%, *τ*^2^ = 0.02, *P* < 0.01).

**Fig. 7:**
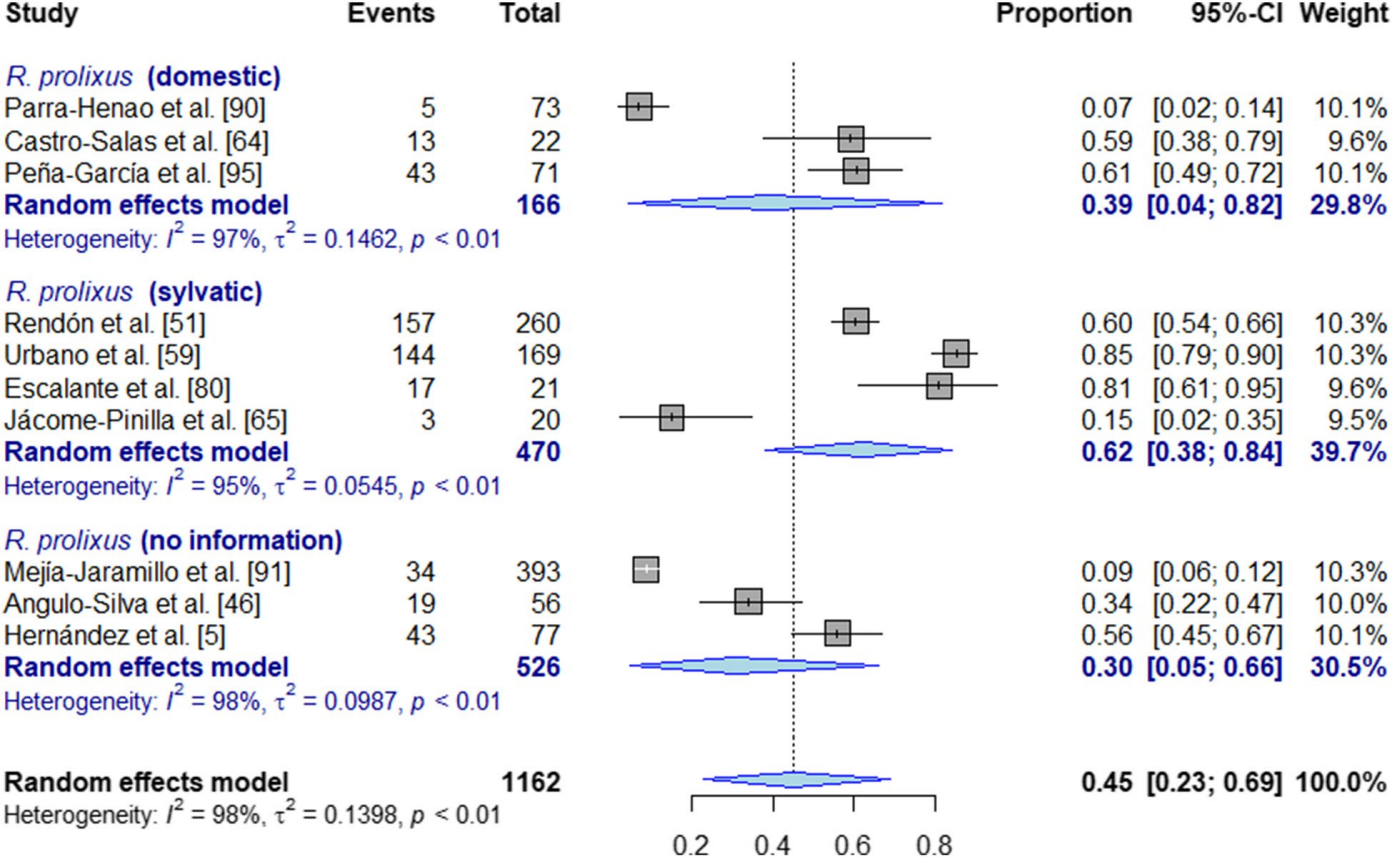
Meta-analysis for pooled prevalence of *T. cruzi* infection using random effects model *for Rhodnius prolixus* by ecotope of collection, and without disaggregation by diagnostic tests used

For the most studied species (*R. prolixus*), 13 studies were found with disaggregated data by ecotope of capture. Although heterogeneity was found similar in both sylvatic and domestic ecotopes (*I*^2^ = 97%, *τ*^2^ = 0.15, *P* < 0.01 and *I*^2^ = 95%, *τ*^2^ = 0.05, *P* < 0.01, respectively), the highest prevalence was obtained for the sylvatic cycle (62.0%, 95% CI: 38–84%; *I*^2^ = 95%, *τ*^2^ = 0.05, *P* < 0.01). Figure 7 shows the meta-analysis for *R. prolixus* by ecotope of collection. Details of all disaggregated results by diagnostic method are presented in Table 5 and further details in Additional file 2: Table S6.

**Table 5:** Results of point or pooled prevalence estimates (meta-analysis using random effects model) for triatomine species by specific and non-specific *T. cruzi* infection in Colombia

Species	Diagnostic subgroup	No. of studies	Triatomine bugs (*n*)	Triatomine bugs (+)	Pooled prevalence (%)	95% CI	*I*^2^ (%)	*τ* ^2^	*P*-value
*B. ferroae*	*T. cruzi*	1	123	0	0	0–1	–		
*B. herreri*	*T. cruzi*	1	44	4	9.09	2–20	–	–	–
*C. pilosa*	*Trypanosoma* spp.	1	28	2	7.1	0–20	–	–	–
*E. cuspidatus*	*T. cruzi*	1	16	9	56.3	31–80	–	–	–
*P. arthuri*	*Trypanosoma* spp.	1	78	0	0	0–2	–	–	–
*P. geniculatus*	*T. cruzi*	1	85	60	70.6	6–8	–	–	–
	*Trypanosoma* spp.	1	368	10	1.7	0–3	–	–	–
*P. rufotuberculatus*	*T. cruzi*	1	86	4	4.7	1–10	–	–	–
	*Trypanosoma* spp.	1	74	1	1.3	0–6	–	–	–
*R. pallescens*	*T. cruzi*	6	317	192	54.0	37–71	86	0.03	<0.01
	*Trypanosoma* spp.	1	66	35	53.0	41–65	–	–	–
*R. pictipes*	*T. cruzi*	1	10	0	0	0–17	–	–	–
	*Trypanosoma* spp.	1	11	1	9.1	0–35	–	–	–
*R. prolixus*	*T. cruzi*	10	1173	485	46.0	23–69	98	0.14	<0.01
	*Trypanosoma* spp.	3	850	71	13.0	3–27	95	0.04	<0.01
*R. prolixus* (domestic)	*T. cruzi*	3	166	61	39.0	4–82	97	0.15	<0.01
	*Trypanosoma* spp.	3	440	44	19.0	1–51	97	0.11	<0.01
*R. prolixus* (sylvatic)	*T. cruzi*	4	470	321	62.0	38–84	95	0.05	<0.01
	*Trypanosoma* spp.	1	170	13	7.6	4–12	–	–	–
*R. prolixus* (peridomestic)	*Trypanosoma* spp.	1	10	0	0	0–17	–	–	–
*R. prolixus* (not specified)	*T. cruzi*	3	526	96	30.0	5–66	98	0.10	<0.01
	*Trypanosoma* spp.	1	220	11	5.0	2–8	–	–	–
*T. dimidiata*	*T. cruzi*	3	392	92	20.0	8–35	90	0.02	<0.01
	*Trypanosoma* spp.	1	2671	222	25.0	0–74	–	–	–
*T. maculata*	*T. cruzi*	4	213	145	68.0	62–74	0	0	<0.0001
	*Trypanosoma* spp.	3	129	16	6.0	0–38	94	0.14	<0.01
*T. venosa*	*Trypanosoma* spp.	1	1115	5	0.004	0–1	–	–	–
Other non-classified triatomines	*T. cruzi*	3	3287	336	27.0	12–46	94	0.04	<0.01
	*Trypanosoma* spp.	1	40	0	0.0	0–4	–	–	–

*Notes: T. cruzi*: includes all *T. cruzi-*specific methods (culture, PCR and mice inoculation); *T.* spp.: includes non-species-specific diagnostic methods (direct observation based on microscopic examination of extruded faeces); +: positive

#### Publication bias and sensitivity analysis

Eggerʼs test was used for cases with at least five studies included for either reservoirs or triatomines. Only one animal reservoir family (Canidae) was found with evidence of potential publication bias. After applying the Trimfill method, estimated prevalence changed from 14.0% (95% CI: 1–37%) to 32.0% (95% CI: 10–58%). Publication bias results are presented in Additional file 2: Table S7 and Figures S2–S4.

On the other hand, sensitivity analysis by repeating all pooled estimates omitting one study at a time did not show a significant change in the estimated pooled prevalence, see Additional file 2: Figures S5, S6 for details.

### Quality assessment

Among the 39 studies included in the meta-analysis, 67% were classified as high-quality, 21% as moderate-quality and 12% as low-quality. The main problems found in the low-quality studies included unclear information on the study period, lack of details on taxonomic classification, and not enough information on selection methods of the subjects. The details are presented in Additional file 2: Tables S8, S9 and Figure S7.

## Discussion

This systematic review obtained 39 studies that included 71 species of potential reservoirs and 18 species of triatomine insects investigated for the presence of *T. cruzi* infection in Colombia. Among them, we found solid evidence of infection in 38 reservoirs and 14 triatomine vector species. To the best of our knowledge, this is the first study that systematically identified this evidence and analysed the heterogeneity of infection rates of *T. cruzi* infection in both animal (non-human) reservoirs and triatomine insects in Colombia.

### Evidence of *T. cruzi* infection in animals in Colombia

Among the 38-mammal species identified as *T. cruzi* reservoirs, 29 belong to four orders exhibiting the highest prevalence: Didelphimorphia (35.0%); Carnivora (17.0%); Rodentia (6.0%); and Chiroptera (15.0%). A subgroup analysis was only possible for Carnivora and Didelphimorphia.

Within the order Didelphimorphia, *D. marsupialis* (common opossum) presented the highest pooled prevalence, 48.0%. The epidemiological importance of this mammal is highlighted for several reasons including its high susceptibility to infection, ability to act as both reservoir and vector, its persistent high infection rates and its highly adaptive behaviour that allows it to live close to both domestic and sylvatic habitats and to transport infection between households [17, 34]. Opossums are mammals with night habits and a clear ability to adapt to artificial ecosystems. In both rural and urban areas, these animals frequently visit houses in search for food, establishing close contact with humans [14, 17, 35]. These animals have been implicated in outbreaks resulting from oral transmission in different areas including in Colombia, suggesting they are able to contaminate human food sources *via* anal secretions [36–38]. The high pooled prevalence found for this species in our study and the already documented tolerance to TcI infection (the same DTU identified in human cases from oral transmission) confirms opossum as the most important species so far identified for transmission to humans in the sylvatic cycle of *T. cruzi* [4, 39, 40].

Our results for the order Carnivora show that *Canis lupus familiaris* presented the highest pooled prevalence (17.0%) in the domestic habitat, with a non-significant difference between serological (24.0%; 95% CI: 1–64%) and non-serologicalal tests (14.0%; 95% CI: 1–31%). Interestingly, diverging results have been found in other countries using parasitological methods [18]. Whereas in the Argentinian Chaco, *Canis lupus familiaris* has been reported as the most important animal in the domestic cycle of *T. cruzi*, with both a high prevalence and a high parasitemia [41, 42], other studies in Brazil have found the domestic dog only with a high seroprevalence, but with a low parasitaemia [43]. It has been suggested that domestic dogs have a paramount role for linking sylvatic and domestic cycles and suggesting that this family could be used as an indicator of recent local transmission [39]. Another interesting contrast occurs with regards to the family Felidae. Species of this family have been reported infected in studies from Argentina and Brazil [44, 45], whereas in our systematic review in Colombia only one study for this family was identified, which reported no *T. cruzi* infection [46].

Another important finding from our review is the estimated pooled prevalence for the orders Chiroptera and Rodentia, 15.0% (95% CI: 5–29%) and 6.0% (95% CI: 2–12%), respectively. The order Chiroptera (to which different species of bats belong) has been reported commonly sharing habitats with triatomines. Their ability to travel long distances and their long lifespan that ranges between 15–50 years [47] make them a very important taxonomic order to keep *T. cruzi* transmission active [48, 49]. Within the Chiroptera, the frugivorous Neotropical bats were the most commonly studied and reported with infected species [50, 51]. Interestingly, it has been documented that *T. cruzi* infection in bats could occur *via* different routes that include oral and congenital transmission, the latter of which could explain the high *T. cruzi* prevalence even in non-insectivorous bats [18].

An additional discrete typing unit identified in bats, TcBat, has been found genetically related to TcI [4]. In recent years, TcBat was isolated from a human case, a five-year-old girl in Colombia [52]. The authors of this case study suggest vectorial or oral as potential infection sources [52]. The high prevalence of *T. cruzi* infection in the order Chiroptera found in our study confirms that species of this order are very relevant reservoirs in sylvatic *T. cruzi* transmission. Additionally, TcBat detected in humans also reveals the potential relevance of Chiroptera to *T. cruzi* human infection. TcBat is not usually investigated *via* molecular diagnostic methods so that the real magnitude of human infection by TcBat still remains unknown [4, 52].

Determining the importance of animal reservoirs in the transmission of *T. cruzi* depends on several factors, such as their ability to be infectious in natural conditions and the concentration of parasites in the blood (parasitemia), which in turn is determined by the species of the animal reservoir, the genotype of *T. cruzi*, the nutritional status of the reservoir, the route of infection and the concomitant infection that ensures its diffusion and maintenance in nature [34]. With regard to rodents, the importance of these animals as *T. cruzi* reservoirs includes their ability to maintain the parasite for long periods of time [53]. Additionally, it has been suggested that rodents act as secondary reservoirs in both domestic and sylvatic habitats where they can play a role in the oral transmission route as part of the food chain to larger predators [18, 54, 55].

### Evidence of *T. cruzi* infection in triatomine insects in Colombia

In Colombia, the triatomine species most efficiently adapted to human habitats are *R. prolixus*, *T. dimidiata* and *T. venosa* [56]. In our study, the pooled prevalence of infection with these species was 46.0% (95% CI: 23–69%), 20.0% (95% CI: 8–35%) and 0.004% (95% CI: 0–1%), respectively.

*Rhodnius prolixus* was the species with the highest number of studies, which allowed us to perform subgroup analysis which evidenced an important difference between sylvatic and domestic cycles, 62.0% (95% CI: 38–84%) and 39.0% (95% CI: 4–82%), respectively. While this triatomine species is the most important domiciliated species reported in Venezuela, Colombian and Central America, our results also highlight the importance of sylvatic colonies. Due to the highly anthropophilic behaviour of this species, its ability to become domiciliated and its high transmission efficiency [57], our results add evidence of the relevance of this species in Colombia, both in domestic and sylvatic cycles. Interestingly, the sylvatic presence of *R. prolixus* has been reported increasingly in both the native palm trees (*Attalea butyracea*) and introduced agro-industrial palms (*Elaeis guineensis*) across the plains of Colombia [51, 58, 59].

The triatomine species with the highest prevalence found in our study was *T. maculata* but only located in the northeast part of the country [60, 61]. In the past, due to its ornithophilic behaviour, it was traditionally considered not very relevant for human transmission [62]. However, recent studies demonstrate their potential importance due to their high prevalence rates and colonisation index [5, 60, 61, 63–65].

Another triatomine species with a very high prevalence was *R. pallescens* with geographical predominance in the north-west part of the country (Caribbean region). This vector has been also associated with *Attalea butyracea* palms [63, 66–87], has been reported frequently in Costa Rica and Nicaragua [67] and it is considered the main vector in Panama [68, 69]. Importantly, this species has been found commonly circulating in areas of oral outbreaks in Colombia [37].

Our study did not find evidence of *T. cruzi* infection in four species (*Belminus ferroae*, *Eratyrus mucronatus*, *Psammolestes arthuri* and *Panstrongylus lignarius*) out of the 18 studied here. *Belminus ferroae* has been considered as not a very relevant vector for human transmission due to its limited hematophagous behaviour [70]. *Eratyrus mucronatus* has been reported in two studies, but it remains unclear whether or not they were found with natural infection [46, 58]. *Psammolestes arthuri* has been reported in two studies associated to nests of birds but with no natural infection [65, 71]. Finally, *P. lignarius* has been reported as infected by *Trypanosoma* spp. based on non-species-specific diagnostic method (microscopy) [71].

Finally, other triatomine species that include R. robustus, R. colombiensis and T. dispar and the animal species *Caluromys lanatus* have been found infected with *T. cruzi* in Colombia and are reported in a study of parasite isolates [33]. However, as that study does not correspond to a prevalence assessment, it was not possible to use those results for the quantitative analyses.

### Public health implications of these results

Several types of dynamic models for Chagas disease have been developed [72]. Most of them have been restricted to domiciliated habitats and in some cases included sylvatic cycle with a single vector and single host. The results from our study show that the heterogeneity of species involved in the sylvatic cycle and the heterogeneity of infection rates among them are worth considering when modelling particularly the sylvatic cycle, as some pioneer studies have started to consider by using multi-host models [73].

Several cultural aspects related to human contact with some of the animal species described in our study could be potentially relevant. Several animal species with clear evidence as *T. cruzi* reservoirs are considered common food sources in indigenous and rural communities. Factors associated with the hunting, sacrifice and manipulation of these animal species could represent a potential risk for accidental contamination with *T. cruzi* in humans. Of particular importance, several species from the order Cingulata (popularly known armadillos and cachicamos) and from the order Chiroptera (bats) are believed to have curative properties in some communities; for this reason their blood is consumed immediately after sacrifice as part of curative rituals, a practice described even from the pre-Columbian era [74, 75].

The results from this study have several potential public health implications. First, to our knowledge, this study is a first baseline, accounting for the diversity of reservoirs and triatomine vectors participating in *T. cruzi* transmission in Colombia, which can help understanding the broader risk to which human populations are exposed to. Secondly, the evidence of *T. cruzi* infection in animal reservoirs from different orders with a lifespan shorter than humans could be considered as an indicator of active transmission in a specific location. Finally, understanding the cultural relations between humans and several wild species studied here could potentially help inform populations on the potential risks associated with certain cultural practices.

### Study limitations

Several relevant limitations arise from this research. First, the lack of sufficient details from several original papers reduces the possibility of more disaggregated analyses for some species. Secondly, our study evidences a high heterogeneity of study designs which makes it more difficult to obtain better pooled estimates [30]. Although publication bias was only detected in one of the species, we are aware that, due to the small number of studies, the test used could be underpowered and the fact that we only found this test as significant for one of the families with the highest number of studies (Canidae) confirms this limitation [31, 32]. Another relevant limitation is that no studies were excluded based on their quality assessment, also due to the very limited number of studies. However, when this sensitivity analysis was possible, we did not find difference between using all or high-quality-only studies. Although we are aware of this limitation, we are also aware of the limitations in conducting studies with animals and particularly in sylvatic areas. The reduced sample size of many of the eligible studies leads to very broad confidence intervals; although this is a limitation, it is also a more reliable measure of the uncertainly in the prevalence estimates from such heterogeneous types of studies.

Finally, an important limitation is related to diagnostic methods. An important recent study in triatomine vectors has identified that optical microscopy used for routine surveillance missed ~50–75% of infections and wrongly scored as infected ~7% of the bugs when only one slide per vector was used [75]. For animals, we found the use of various tests with high specificity for *T. cruzi* detection such as xenodiagnostic, haemoculture and coloured smears, and in the case of the family Canidae serological methods were used and in some cases with PCR confirmation [76, 77]. For vectors, however, most of the studies were conducted by direct microscopic observation which, as discussed above, is not highly species-specific. In the more recent studies PCR has also been included which certainly improves the quality of the measurement. We acknowledge that the use of optic microscopy can lead primarily to an underestimate of the prevalence infection rates in vectors. However, due the lack of data on the operative performance (sensitivity and specificity) of some of the techniques used in the original studies which can vary between years and operators, unfortunately we could not adjust the prevalence estimates in our meta-analysis. We have conducted subgroup analysis by diagnostic techniques in order to address this issue in vectors (see Table 5). This analysis suggests that techniques such as PCR (that can also differentiate species) are significantly more sensitive and able to detect highest values of the prevalence.

## Conclusions

To our knowledge, this is the first systematic evaluation and quantitative analysis of *T. cruzi* infection rates in triatomine insects and potential animal reservoirs in Colombia. The results also confirm solid evidence of the presence of *T. cruzi* infection in 38 out of 71 animal species and in 14 out of 18 triatomine species studied. Among animals, the species with the highest pooled prevalence were opossums (*Didelphis marsupialis*) and domestic dogs (*Canis lupus familiaris*). Among triatomines, the highest prevalence was found in *T. maculata* from peridomestic ecotopes followed by *R. prolixus* and *R. pallescens*, both from sylvatic ecotopes. Further research is needed to better understand the interaction between these species, humans and parasites.

## Additional files

**Additional file 1.** PRISMA checklist.

**Additional file 2.** **Text S1.** Additional text on quality assessment. **Table S1.** Boolean algorithms for literature search as of 5th of April 2018. **Table S2.** Pooled prevalence estimates of *T. cruzi* infection in both triatomines and potential animal reservoirs by departments (firs administrative units) in Colombia. **Table S3**. Detailed data for the 18 studies chosen for potential *T. cruzi* reservoirs in Colombia. **Table S4.** Detailed data for the 28 studies chosen for *Trypanosoma cruzi* triatomine vectors in Colombia. **Table S5.** Results of point or pooled prevalence estimates (meta-analysis using random effects model) by detailed diagnostic methods for the different orders of potential *Trypanosoma cruzi* animal reservoirs studied in Colombia. **Table S6.** Results of point or pooled prevalence estimates (meta-analysis using random effects model) of *T. cruzi* infection by detailed diagnostic methods for the different orders of triatomines studied in Colombia. **Table S7.** Results of Eggerʼs test for publication bias assessment for estimates of *T. cruzi* infection rates in animal reservoirs and triatomine vectors in Colombia. **Table S8.** Results of point or pooled prevalence estimates (meta-analysis using random effects model) for the different orders of potential *Trypanosoma cruzi* reservoirs studied in Colombia, using only high-quality reports. **Table S9.** Results of point or pooled prevalence estimates (meta-analysis using random effects model) of *T. cruzi* infection for the different orders of triatomines studied in Colombia, using only high-quality reports. **Figure S1.** Content quality assessment of the 39 studies used in the meta-analysis. **Figure S2.** Funnel plot of the 95% CI pseudo limits for the estimates of *T. cruzi* prevalence in animal reservoirs in Colombia.

## Data Availability

The datasets generated and analysed during the present study are available in a public repository, github.com/zmcucunuba/heterogeneity-tcruzi-review.

## Abbreviations

95% CI: 95% confidence interval
PCR: polymerase chain reaction
