# Supplementary material for: Heterogeneity of Trypanosoma cruzi infection rates in vectors and animal reservoirs in Colombia: a systematic review and meta-analysis
Source: Parasit Vectors. 2019 Jun 20;12:308. doi: 10.1186/s13071-019-3541-5 (PMC6585012; doi:10.1186/s13071-019-3541-5)
Supplement: Supplementary file 2 — Additional file 2. Text S1. Additional text on quality assessment. Table S1. Boolean algorithms for literature search as of 5th of April 2018. Table S2. Pooled prevalence estimates of T. cruzi infection in both triatomines and potential animal reservoirs by departments (firs administrative units) in Colombia. Table S3. Detailed data for the 18 studies chosen for potential T. cruzi reservoirs in Colombia. Table S4. Detailed data for the 28 studies chosen for Trypanosoma cruzi triatomine vectors in Colombia. Table S5. Results of point or pooled prevalence estimates (meta-analysis using random effects model) by detailed diagnostic methods for the different orders of potential Trypanosoma cruzi animal reservoirs studied in Colombia. Table S6. Results of point or pooled prevalence estimates (meta-analysis using random effects model) of T. cruzi infection by detailed diagnostic methods for the different orders of triatomines studied in Colombia. Table S7. Results of Eggerʼs test for publication bias assessment for estimates of T. cruzi infection rates in animal reservoirs and triatomine vectors in Colombia. Table S8. Results of point or pooled prevalence estimates (meta-analysis using random effects model) for the different orders of potential Trypanosoma cruzi reservoirs studied in Colombia, using only high-quality reports. Table S9. Results of point or pooled prevalence estimates (meta-analysis using random effects model) of T. cruzi infection for the different orders of triatomines studied in Colombia, using only high-quality reports. Figure S1. Content quality assessment of the 39 studies used in the meta-analysis. Figure S2. Funnel plot of the 95% CI pseudo limits for the estimates of T. cruzi prevalence in animal reservoirs in Colombia. [file 13071_2019_3541_MOESM2_ESM.docx]

**Additional file 2. F****urther details on Methods, Results and Analyses.**

**Heterogeneity in *Trypanosoma cruzi* infection in triatomine vectors and animal reservoirs in Colombia: a systematic review and meta-analysis**

Eliana Rodríguez-Monguí^1^, Omar Cantillo-Barraza ^2^, Franklin-Edwin Prieto^1^, Zulma M. Cucunubá^3^

1. Facultad de Medicina, Universidad El Bosque, Bogotá, Colombia.
2. Laboratorio Biología y Control de Enfermedades Infecciosas (BCEI), Universidad de Antioquia, Medellín, Colombia.
3. Department of Infection Disease Epidemiology, Imperial College London, London, United Kingdom.

Authors e-mail addresses:

Eliana Rodríguez-Monguí eliana.rodmon@gmail.com

Omar Cantillo-Barraza [omarcantillo@gmail.com](mailto:omarcantillo@gmail.com)

Franklin Prieto [franklynprieto@yahoo.com](mailto:franklynprieto@yahoo.com)

Zulma M. Cucunubá [zulma.cucunuba@imperial.ac.uk](mailto:zulma.cucunuba@imperial.ac.uk)

Corresponding author: Zulma M. Cucunubá, Department of Infection Disease Epidemiology, Imperial College London. zulma.cucunuba@imperial.ac.uk

[**Additional Text on Quality Assessment** 2](#_Toc520732894)

[**Additional Tables** 3](#_Toc520732895)

[**Additional Figures** 21](#_Toc520732896)

#

# **Text S1.** **Additional text on Quality Assessment**

An evaluation matrix was designed for content quality assessment [1], where the following items were examined:

- Was the research question/objective clearly described and stated?
- Was the sampling method described in detail?
- Was the period of study clearly stated?
- Was the serological test method clearly pointed out?
- Were the subjects categorized into different subgroups?

To each item, a score was given based in these criteria:


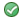
2= low risk "yes"


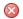
 0= high risk "no"


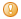
 1= unclear risk "unsure"

Did the study address a clearly described and focused question / problem?

The method of selection of the subjects described in detail, including the protocol method of search and capture of triatomine bugs and reservoirs (study design), is it appropriate to answer the research question? (selection and information bias)

Was the diagnostic test method for *T. cruzi* infection in vectors and / or animal reservoirs clearly informed and adequate to answer the research question? (information bias)

Was the study period clearly established? (selection bias)

 Were the subjects categorized into different subgroups; Could there be confounding factors that have not been taken into account? (confusion)

**Table S1. Boolean algorithms for literature search as of 5^th^ of April 2018.**

| Database | Algorithm | Number of titles | Filter |
| --- | --- | --- | --- |
| Web of knowledge | (((trypanosomiasis OR trypanosoma cruzi OR Chagas disease) AND (transmission OR cycle OR ecoepidemiology OR ecology OR triatominae OR vector OR reservoir OR mammals*) AND colombia)) | 189 | None |
| PubMed | ("trypanosomiasis"[MeSH Terms] OR ("trypanosoma cruzi"[MeSH Terms] OR ("trypanosoma"[All Fields] AND "cruzi"[All Fields]) OR "trypanosoma cruzi"[All Fields]) OR "trypanosomiasis"[All Fields] OR "Chagas disease"[All Fields] OR "Chagas disease"[MeSH Terms]) |  |  |
| LILACS | (tw:((Chagas Disease OR Enfermedad de Chagas OR Doença de Chagas OR Trypanosomiasis OR Tripanosomiasis OR Tripanossomíase OR Trypanosoma cruzi))) AND (tw:(Colombia OR Colômbia )) AND tw:(transmission OR transmisión OR transmissão OR Disease Vectors OR Vectores de Enfermedades OR Vetores de Doenças OR Triatominae OR Triatominae OR Triatominae OR mammals OR mamíferos)) | 290 | None |
| EMBASE | 1. 'trypanosoma cruzi'/exp OR 'trypanosoma cruzi'  2. 'chagas disease'/exp OR 'chagas disease'  3. 'trypanosomiasis'  4. #1 OR #2 OR #3  5. 'disease transmission'  6. 'disease carrier'  7. 'triatominae'  8. 'ecology'  9. cycle AND transmission  10. 'mammal'  11. #5 OR #6 OR #7 OR #8 OR #9 OR #10  12. #4 AND #11  13. 'colombia'  14. #12 AND #13 | 227 | None |
| Google  Scholar | NA | 3 | None |
| Digital Repository National university | NA | 4 | None |
| Total | | 960 |  |

**Table S2. Pooled prevalence estimates of *Trypanosoma cruzi* infection in both triatomines and potential animal reservoirs by departments (firs administrative units) in Colombia**

| Locations | Number of studies | Reservoirs (n) | Reservoirs (+) | Pooled Prevalence  (%) | 95%CI | I^2^  (%) | τ^2^ | p-Value |
| --- | --- | --- | --- | --- | --- | --- | --- | --- |
|  |  |  |  |  |  |  |  |  |
| Potential Reservoirs | | | | | | | | |
| Various* | 2 | 20228 | 983 | 9.0 | (2-22) | 98 | 0.02 | <0.01 |
| Antioquia | 1 | 70 | 5 | 7.1 | (2-15) | ---- | ---- | ---- |
| Boyacá | 2 | 140 | 34 | 17.0 | (0-57) | 80 | 0.01 | 0.03 |
| Bolívar | 2 | 294 | 205 | 75.0 | (58-89) | 72 | 0.01 | 0.06 |
| Casanare | 3 | 387 | 75 | 14.0 | (2-32) | 94 | 0.03 | <0.01 |
| Córdoba | 2 | 99 | 14 | 17.0 | (0-57) | 94 | 0.09 | <0.01 |
| Guajira | 1 | 12 | 2 | 16.6 | (0-44) | ---- | ---- | ---- |
| Meta | 2 | 999 | 142 | 17.0 | (5-34) | 97 | 0.02 | <0.01 |
| Magdalena | 1 | 115 | 20 | 17.4 | (11-25) | ---- | ---- | ---- |
| Nariño | 1 | 325 | 24 | 7.4 | (5-11) | ---- | ---- | ---- |
| SNSM** | 1 | 151 | 72 | 48.0 | (40-56) | ---- | ---- | ---- |
| Triatomine bugs | | | | | | | | |
| Antioquia | 1 | 124 | 12 | 11.0 | (6-18) | ---- | ---- | ---- |
| Atlántico | 1 | 12 | 0 | 0.0 | (0-14) | ---- | ---- | ---- |
| Arauca | 1 | 29 | 13 | 44.8 | (27-63) | ---- | ---- | ---- |
| Bolívar | 4 | 244 | 168 | 68.8 | (61-76) | 34 | 0.00 | 0.21 |
| Boyacá | 1 | 4579 | 197 | 4.3 | (0-14) | ---- | ---- | ---- |
| Caquetá | 1 | 18 | 9 | 50.0 | (27-73) | ---- | ---- | ---- |
| Casanare | 5 | 604 | 351 | 46.0 | (23-71) | 97 | 0.08 | <0.01 |
| Cesar | 3 | 3286 | 295 | 24.0 | (0-65) | 99 | 0.14 | <0.01 |
| Córdoba | 1 | 22 | 18 | 81.8 | (63-96) | ---- | ---- | ---- |
| Guajira | 3 | 444 | 128 | 42.0 | (13-76) | 97 | 0.08 | <0.01 |
| Magdalena | 3 | 167 | 96 | 58.0 | (50-65) | 0 | 0.00 | 0.61 |
| Meta | 1 | 384 | 51 | 13.2 | (10-17) | ---- | ---- | ---- |
| Norte de Santander | 1 | 123 | 0 | 0.0 | (0-1) | ---- | ---- | ---- |
| Santander | 1 | 17 | 9 | 52.9 | (29-76) | ---- | ---- | ---- |
| Vichada | 1 | 28 | 18 | 64.2 | (45-81) | ---- | ---- | ---- |
| SNSM | 3 | 740 | 67 | 9.1 | (7-11) | 0 | 0.00 | 0.89 |
| Various* | 2 | 470 | 221 | 46.0 | (18-75) | 98 | 0.05 | <0.01 |

*Non-disaggregated results

**SNSM: Sierra Nevada de Santa Marta

**Table S3. Detailed data for the 18 studies chosen for potential *Trypanosoma cruzi* reservoirs in Colombia**

| **Reference** | **Study period** | **Departament**  **(first administrative unit)** | **Order** | **Species** | **No. of animals** | **No. of infected animals** | **Prevalence for**  **order**  **%** | **Diagnostic methods** | **Quality Score** | **Ref** |
| --- | --- | --- | --- | --- | --- | --- | --- | --- | --- | --- |
| D'Alessandro 1971 | 1968 | Casanare,Vichada,Meta | Artiodactyla | *O. virginianus* | 9 | 2 | 27.27 | Non-serological | 3 | [2] |
|  |  |  |  | *M. gouazoubira medemi* | 2 | 1 |  |  |  |  |
| Marinkelle 1982 (a) | 1961-1972 | Colombia | Primates | ---- | 343 | 53 | 15.45 | Non-serological | 5 | [3] |
| Marinkelle 1982 (b) | 1961-1972 | Colombia | Chiroptera | ---- | 19885 | 930 | 4.67 | Non-serological | 9 | [4] |
| D'Alessandro et al. 1984 | 1978-1979 | Meta | Cingulata | *D. kappleri* | 7 | 2 | 19.04 | Non-serological | 8 | [5] |
|  |  |  |  | *D. sabanicola* | 10 | 1 |  |  |  |  |
|  |  |  |  | *P. giganteus* | 4 | 1 |  |  |  |  |
|  |  |  | Pilosa | *M. tridactyla* | 1 | 0 | 16.66 |  |  |  |
|  |  |  |  | *T. tetradactyla* | 5 | 1 |  |  |  |  |
|  |  |  | Didelphimorphia | *D. marsupialis* | 62 | 25 | 18.97 |  |  |  |
|  |  |  |  | *Marmosa s.p.* | 4 | 1 |  |  |  |  |
|  |  |  |  | *M. murina* | 32 | 0 |  |  |  |  |
|  |  |  |  | *M. nudicaudatus* | 37 | 0 |  |  |  |  |
|  |  |  |  | *Monodelphis s.p.* | 1 | 0 |  |  |  |  |
|  |  |  |  | *L. crassicaudata* | 1 | 0 |  |  |  |  |
|  |  |  | Carnivora | *C. thous* | 6 | 0 | 18.75 |  |  |  |
|  |  |  |  | *F. concolor* | 1 | 0 |  |  |  |  |
|  |  |  |  | *F. pardalis* | 2 | 0 |  |  |  |  |
|  |  |  |  | *P. flavus* | 7 | 3 |  |  |  |  |
|  |  |  | Rodentia | *C. porcellus* | 1 | 0 | 8.63 |  |  |  |
|  |  |  |  | *H. hydrochaeris* | 57 | 5 |  |  |  |  |
|  |  |  |  | *R. rattus* | 1 | 0 |  |  |  |  |
|  |  |  |  | *Z. brevicauda* | 43 | 0 |  |  |  |  |
|  |  |  |  | *N. spinosus* | 7 | 0 |  |  |  |  |
|  |  |  |  | *N. squamipes* | 19 | 0 |  |  |  |  |
|  |  |  |  | *S. alstoni* | 1 | 0 |  |  |  |  |
|  |  |  |  | *Coendou s.p* | 1 | 0 |  |  |  |  |
|  |  |  |  | *Proechymis s.p* | 224 | 23 |  |  |  |  |
|  |  |  |  | *O. concolor* | 15 | 1 |  |  |  |  |
|  |  |  |  | *O. delicates* | 1 | 0 |  |  |  |  |
|  |  |  |  | *O. fulvescens* | 11 | 0 |  |  |  |  |
|  |  |  |  | *C. paca* | 74 | 10 |  |  |  |  |
|  |  |  |  | *D. fuliginosa* | 8 | 1 |  |  |  |  |
|  |  |  | Chiroptera | *Artibeus s.p.* | 11 | 1 | 3.84 |  |  |  |
|  |  |  |  | *A. jamaicensis* | 2 | 1 |  |  |  |  |
|  |  |  |  | *P. discolour* | 2 | 0 |  |  |  |  |
|  |  |  |  | *E. furinalis* | 3 | 1 |  |  |  |  |
|  |  |  |  | *L. borealis* | 1 | 0 |  |  |  |  |
|  |  |  |  | *M. nigricans* | 2 | 0 |  |  |  |  |
|  |  |  |  | *E. perotis* | 1 | 0 |  |  |  |  |
|  |  |  |  | *M. major* | 33 | 0 |  |  |  |  |
|  |  |  |  | *M. ater* | 20 | 1 |  |  |  |  |
|  |  |  |  | *N. labialis* | 29 | 0 |  |  |  |  |
|  |  |  | Artiodactyla | *O. virginianus* | 6 | 2 | 22.22 |  |  |  |
|  |  |  |  | *Tayassu s.p.* | 2 | 0 |  |  |  |  |
|  |  |  |  | *T. albirostris* | 1 | 0 |  |  |  |  |
| Travi et al. 1994 | 1991 | Narino, Cordoba | Cingulata | *D. novemcinctus* | 1 | 1 | 100 | Non-serological | 4 | [6] |
|  |  |  | Pilosa | *B. variegatus* | 45 | 0 | 0 |  |  |  |
|  |  |  |  | *C. hoffmanni* | 1 | 0 |  |  |  |  |
|  |  |  |  | *C. Didactylus* | 4 | 0 |  |  |  |  |
|  |  |  |  | *T. tetradactyla* | 7 | 0 |  |  |  |  |
|  |  |  | Didelphimorphia | *D. marsupialis* | 40 | 16 | 26.22 |  |  |  |
|  |  |  |  | *P. opossum* | 21 | 0 |  |  |  |  |
|  |  |  | Carnivora | *P. flavus* | 2 | 1 | 50.0 |  |  |  |
|  |  |  | Rodentia | *R. rattus* | 87 | 4 | 3.29 |  |  |  |
|  |  |  |  | *T. mirae* | 23 | 3 |  |  |  |  |
|  |  |  |  | *P. semiespinosus* | 163 | 2 |  |  |  |  |
| Wolff et al. 2001 | 1997-1999 | Antioquia | Cingulata | *D. novemcinctus* | 10 | 1 | 20.0 | Non-serological | 9 | [7] |
|  |  |  | Didelphimorphia | *D. marsupialis* |  | 1 |  |  |  |  |
|  |  |  |  | *P. opossum* |  | 0 |  |  |  |  |
|  |  |  |  | *Marmosa cf. Robinsoni* |  | 0 |  |  |  |  |
|  |  |  | Rodentia | *H. gimnurus* |  | 0 |  |  |  |  |
|  |  |  |  | *Proechymis sp* |  | 0 |  |  |  |  |
|  |  |  |  | *Oryzomis sp* |  | 0 |  |  |  |  |
|  |  |  | Carnivora | *C. lupus* | 60 | 3 | 5.0 | Serological |  |  |
| Manrique et al.2012 | 2009 | Boyaca | Carnivora | *C. lupus* | 60 | 9 | 15.0 | serological | 7 | [8] |
| Ramírez et al. 2013 | 2012 | Boyaca | Carnivora | *C. lupus* | 80 | 25 | 31.25 | Non-serological | 7 | [9] |
| Ramírez et al. 2014 | 2010 | Casanare | Chiroptera | *A. planirostris* | 16 | 55 | 31.42 | Non-serological | 5 | [10] |
|  |  |  |  | *A. fuliginosus* | 18 |  |  |  |  |  |
|  |  |  |  | *C. perspicillata* | 83 |  |  |  |  |  |
|  |  |  |  | *D. rotundus* | 22 |  |  |  |  |  |
|  |  |  |  | *M. oxyotus* | 23 |  |  |  |  |  |
|  |  |  |  | *R. naso* | 13 |  |  |  |  |  |
| Vásquez et al. 2013 | 2003-2004 | Momposina | Carnivora | *C. lupus* | 66 | 10 | 15.15 | mixed | 9 | [11] |
|  |  |  | Cingulata | *D. novemcinctus* | 3 | 1 | 33.33 |  |  |  |
|  |  |  | Pilosa | *V. gryphus* | 4 | 1 | 25.0 |  |  |  |
|  |  |  | Rodentia | *M. musculus* | 1 | 0 | 0 |  |  |  |
|  |  |  |  | *Rattus sp* | 1 | 0 |  |  |  |  |
|  |  |  |  | *D. punctate* | 3 | 0 |  |  |  |  |
|  |  |  | Primate | *A. caraya* | 1 | 0 | 0 |  |  |  |
|  |  |  | Lagomorpha | *O. cuniculus* | 9 | 2 | 22.22 |  |  |  |
|  |  |  | Chiroptera | *Glossophaga s.p* | 4 | 2 | 18.51 |  |  |  |
|  |  |  |  | *Sturnira s.p* | 1 | 0 |  |  |  |  |
|  |  |  |  | *M. nigricans* | 4 | 0 |  |  |  |  |
|  |  |  |  | *Molossus s.p* | 6 | 2 |  |  |  |  |
|  |  |  |  | *M. molossus* | 4 | 0 |  |  |  |  |
|  |  |  |  | *M. bondae* | 7 | 1 |  |  |  |  |
|  |  |  |  | *N. leporinus* | 1 | 0 |  |  |  |  |
| Peña-García et al. 2014 | 2010-2011 | Guajira | Didelphimorphia | *D. marsupialis* | 1 | 1 | 100 | Non-serological | 10 | [12] |
|  |  |  |  | *M. nudicaudatus* | 1 | 1 |  |  |  |  |
|  |  |  | Carnivora | *C. lupus* | 10 | 0 | 0 |  |  |  |
| Cantillo-Barraza et al. 2014 | 2011 | Isla margarita | Didelphimorphia | *D. marsupialis* | 22 | 19 | 86.95 | Non-serological | 7 | [13] |
|  |  |  |  | *Marmosa cf. Robinsoni* | 1 | 1 |  |  |  |  |
|  |  |  | Rodentia | *R. rattus* | 1 | 0 | 75.0 |  |  |  |
|  |  |  |  | *Oecomys s.p* | 1 | 1 |  |  |  |  |
|  |  |  |  | *Z. brunneus* | 1 | 1 |  |  |  |  |
|  |  |  |  | *Diplomys s.p* | 1 | 1 |  |  |  |  |
| Mejia-Jaramillo et al. 2014 | 2011 | Sierra Nevada de Santa Marta | Didelphimorphia | *D. marsupialis* | 2 | 1 | 50.0 | Non-serological | 6 | [14] |
|  |  |  | Carnivora | *C. lupus* | 126 | 63 | 49.61 |  |  |  |
|  |  |  |  | *F. silvestris catus* | 5 | 2 |  |  |  |  |
|  |  |  | Rodentia | *C. porcellus* | 3 | 0 | 35.29 |  |  |  |
|  |  |  |  | *H. anomalus* | 7 | 3 |  |  |  |  |
|  |  |  |  | *P. semiespinosus* | 6 | 3 |  |  |  |  |
|  |  |  |  | *D. punctate* | 1 | 0 |  |  |  |  |
|  |  |  | Artiodactyla | *S. scrofa* | 1 | 0 | 0 |  |  |  |
| Rendón et al. 2015 | 2012 | Casanare | Pilosa | *T. tetradactyla* | 2 | 2 | 100 | Non-serological | 7 | [15] |
|  |  |  | Didelphimorphia | *D. marsupialis* | 24 | 5 | 13.15 |  |  |  |
|  |  |  |  | *M. andersoni* | 52 | 5 |  |  |  |  |
|  |  |  | Carnivora | *C. lupus* | 17 | 0 | 0 |  |  |  |
|  |  |  | Rodentia | Undentified | 23 | 4 | 11.11 |  |  |  |
|  |  |  |  | *H. anomalus* | 2 | 0 |  |  |  |  |
|  |  |  |  | *P. oconnelli* | 9 | 0 |  |  |  |  |
|  |  |  |  | *D. fuliginosa* | 2 | 0 |  |  |  |  |
|  |  |  | Chiroptera | Undentified | 39 | 2 | 6.52 |  |  |  |
|  |  |  |  | *A. lituratus* | 7 | 1 |  |  |  |  |
| Cantillo-Barraza et al. 2015 | 2010-2012 | Isla margarita | Didelphimorphia | *D. marsupialis* | 13 | 8 | 61.53 | serological | 10 | [16] |
|  |  |  | Carnivora | *C. lupus* | 244 | 174 | 71.31 |  |  |  |
|  |  |  | Rodentia | *R. norvegicus* | 10 | 0 | 0 |  |  |  |
| Angulo-Silva et al. 2016 | 2008 | Casanare | Carnivora | *C. lupus* | 24 | 1 | 2.85 | Non-serological | 7 | [17] |
|  |  |  |  | *F. silvestris catus* | 11 | 0 |  |  |  |  |
| Delgado-Sarmiento et al. 2016 | 2013 | Cordoba | Chiroptera | *Carolia perspicillata* y *Dermanura phaeotis* (frugívoro) y *Molossus molossus* (insectívoro) | 30 | 11 | 36.66 | Non-serological | 5 | [18] |
| Zuleta-Dueñas et al. 2017 | 2014 | Casanare | Didelphimorphia | *D. marsupialis* | 2 | 2 | 100 | Non-serological | 9 | [19] |
|  |  |  | Carnivora | *C. lupus* | 2 | 1 | 50.0 |  |  |  |
| Jaimes-Dueñez et al. 2017 | 2015 | Meta | Carnivora | *C. lupus* | 242 | 62 | 25.61 | mixed | 9 | [20] |
| Parra et al. 2004 | 1999 | Sierra Nevada de Santa Marta | Carnivora | *C. lupus* | 1 | 1 | 100 | Non-serological | 8 | [21] |
| Sotoet al. 2014 | 2010 | Cesar | Didelphimorphia | *D. marsupialis* | 2 | 1 | 50.0 | Non-serological | 7 | [22] |
| Reyes et al. 2017 | 2014 | Santander | Didelphimorphia | *D. marsupialis* | 1 | 1 | 100 | Non-serological | 9 | [23] |

Non-serological: includes parasitological methods (blood smears hemoculture and xenodiagnostic) and molecular methods (PCR).

Serological: includes ELISA, Immunofluorescence Tests (IFAT) or Rapid Tests based on whole parasite or recombined antigens.

**Table S4. Detailed data for the 28 studies chosen for *Trypanosoma cruzi* triatomine vectors in Colombia**

| **Reference** | **Study period** | **Department** | **Genus** | **Species** | **No. of Triatomine bugs** | **No. of Triatomine bugs infected** | **Diagnostic methods** | **Quality Score** | **Ref** |
| --- | --- | --- | --- | --- | --- | --- | --- | --- | --- |
| D'Alessandro et al. 1984 | 1978-1979 | Meta | *Rhodnius* | *R. prolixus* | 254 | 42 | No species-specific | 8 | [5] |
|  |  |  | *Panstrongylus* | *P. geniculatus* | 2 | 2 |  |  |  |
|  |  |  | *Panstrongylus* | *P. lignarius* | 8 | 5 |  |  |  |
|  |  |  | *Triatoma* | *T. maculate* | 14 | 0 |  |  |  |
|  |  |  | *Cavernicola* | *C. pilosa* | 28 | 2 |  |  |  |
|  |  |  | *Psammolestes* | *P. arthuri* | 78 | 0 |  |  |  |
| Wolff et al. 2001 | 1997-1999 | Antioquia | *Panstrongylus* | *P. rufotuberculatus* | 86 | 4 | Species-specific | 9 | [7] |
|  |  |  | varios | *P.geniculatus,T.dispar* | 38 | 10 |  |  |  |
| Sandoval et al. 2004 | 2001 | Cesar | *Belminus* | *B. herreri* | 44 | 4 | Species-specific | 9 | [24] |
| Parra et al. 2004 | 1999 | Sierra Nevada de Santa Marta | *Rhodnius* | *R. prolixus* | 30 | 6 | No species-specific | 8 | [21] |
|  |  |  | varios | *R.prolixus, T.dimidiata, P. geniculatus, T. maculata* | 40 | 0 |  |  |  |
| Cortéz et al. 2005 | 2003 | Bolivar | *Triatoma* | *T. maculata* | 28 | 16 | No species-specific | 9 | [24] |
|  |  |  | *Eratyrus* | *E. cuspidatus* | 2 | 0 |  |  |  |
| Dib 2009 | 2006 | Magdalena | *Rhodnius* | *R. pallescens* | 10 | 8 | Species-specific | 7 | [25] |
|  |  |  | *Panstrongylus* | *P. geniculatus* | 6 | 5 |  |  |  |
|  |  |  | *Triatoma* | *T. maculate* | 3 | 0 |  |  |  |
|  |  |  | *Eratyrus* | *E. cuspidatus* | 16 | 9 |  |  |  |
| Parra-Henao et al. 2009 | 2006-2008 | Cesar, Guajira, Magdalena | *Rhodnius* | *R. prolixus* | 73 | 5 | Species-specific | 9 | [26] |
|  |  |  | *Rhodnius* | *R. pallescens* | 11 | 0 |  |  |  |
|  |  |  | *Panstrongylus* | *P. geniculatus* | 3 | 0 |  |  |  |
|  |  |  | *Triatoma* | *T. maculata* | 9 | 0 |  |  |  |
|  |  |  | *Triatoma* | *T. dimidiata* | 111 | 12 |  |  |  |
| Cantillo-Barraza et al. 2010 | 2006-2008 | Isla margarita | *Rhodnius* | *R. pallescens* | 2 | 2 | Species-specific | 10 | [27] |
|  |  |  | *Triatoma* | *T. maculata* | 41 | 27 |  |  |  |
| Sandoval et al. 2010 | 2004-2006 | Norte de Santander | *Belminus* | *B. ferroae* | 123 | 0 | Species-specific | 8 | [28] |
| Montilla et al. 2011 | 2006 y 2007 | Cesar | varios | *R.prolixus, T.maculata, T.dimidiata* | 3107 | 280 | Species-specific | 8 | [29] |
| Angulo et al. 2012 | 2003-2006 | Llanos Orientales (Casanare-Arauca) | various | Various | 113 | 33 | Species-specific | 7 | [30] |
| Vásquez et al. 2013 | 2003-2004 | Momposina | *Rhodnius* | *R. pallescens* | 66 | 39 | Species-specific | 9 | [11] |
| Castro-Salaset al. 2013 | 2010 | Vichada | *Rhodnius* | *R. prolixus* | 24 | 15 | Species-specific | 10 | [31] |
|  |  |  | *Panstrongylus* | *P. geniculatus* | 2 | 2 |  |  |  |
|  |  |  | *Triatoma* | *T. maculate* | 2 | 1 |  |  |  |
| Parra-Henao et al. 2015 | 2000,2008,2010,2013 | Atlantico, Boyaca, Caqueta, Guajira | *Panstrongylus* | *P. geniculatus* | 368 | 10 | No species-specific | 4 | [32] |
|  |  |  | *Eratyrus* | *E. cuspidatus* | 1 | 0 |  |  |  |
|  |  |  | *Eratyrus* | *E. mucronatus* | 1 | 0 |  |  |  |
|  |  |  | *Rhodnius* | *R. prolixus* | 566 | 20 |  |  |  |
|  |  |  | *Rhodnius* | *R. pictipes* | 12 | 1 |  |  |  |
|  |  |  | *Rhodnius* | *R. pallescens* | 2 | 0 |  |  |  |
|  |  |  | *Rhodnius* | *Rhodnius spp* | 31 | 0 |  |  |  |
|  |  |  | *Panstrongylus* | *P. rufotuberculatus* | 74 | 1 |  |  |  |
|  |  |  | *Panstrongylus* | *Panstrongyluss spp* | 12 | 0 |  |  |  |
|  |  |  | *Triatoma* | *T. maculate* | 87 | 0 |  |  |  |
|  |  |  | *Triatoma* | *T. dimidiate* | 2671 | 222 |  |  |  |
|  |  |  | *Triatoma* | *T. venosa* | 1115 | 5 |  |  |  |
|  |  |  | *Triatoma* | *Triatoma spp* | 3 | 0 |  |  |  |
| Peña-García et al. 2014 | 2010-2011 | Guajira | *Rhodnius* | *R. prolixus* | 71 | 43 | Species-specific | 10 | [12] |
|  |  |  | *Triatoma* | *T. dimidiate* | 7 | 0 |  |  |  |
| Gómez-Melendro et al. 2014 | 2013 | Guajira | *Triatoma* | *T. maculata* | 32 | 19 | Species-specific | 9 | [33] |
| Mejia-Jaramillo et al. 2014 | 2011 | Sierra Nevada de Santa Marta | *Rhodnius* | *R. prolixus* | 393 | 34 | Species-specific | 7 | [14] |
|  |  |  | *Rhodnius* | *R. pictipes* | 10 | 0 |  |  |  |
|  |  |  | *Panstrongylus* | *P. rufotuberculatus* | 2 | 0 |  |  |  |
|  |  |  | *Panstrongylus* | *P. geniculatus* | 2 | 0 |  |  |  |
|  |  |  | *Triatoma* | *T. dimidiate* | 56 | 10 |  |  |  |
| Soto et al. 2014 | 2010 | Cesar | *Rhodnius* | *R. pallescens* | 135 | 84 | Species-specific | 7 | [22] |
| Rendón et al. 2015 | 2012 | Casanare | *Rhodnius* | *R. prolixus* | 269 | 162 | Species-specific | 7 | [15] |
| Urbano et al. 2015 | 2012 | Casanare | *Rhodnius* | *R. prolixus* | 169 | 144 | Species-specific | 10 | [34] |
| Escalante et al. 2015 | 2013 | Region Caribe: Atlantico, Bolivar, Cordoba, Sucre | *Panstrongylus* | *P. geniculatus* | 9 | 6 | Species-specific | 9 | [35] |
|  |  |  | *Rhodnius* | *R. pallescens* | 58 | 44 |  |  |  |
|  |  |  | *Rhodnius* | *R. prolixus* | 21 | 17 |  |  |  |
|  |  |  | *Triatoma* | *T. dimidiate* | 1 | 1 |  |  |  |
| Cantillo-Barraza et al. 2015 | 2010-2012 | Isla margarita | *Rhodnius* | *R. pallescens* | 6 | 3 | Species-specific | 10 | [35] |
|  |  |  | *Triatoma* | *T. maculate* | 106 | 76 |  |  |  |
|  |  |  | *Eratyrus* | *E. cuspidatus* | 1 | 0 |  |  |  |
| Parra et al. 2015 | 2007-2008 | SNSM, Antioquia, Magdalena, Santander, Boyacá, Huila, Bolívar y Sucre | *Triatoma* | *T. dimidiate* | 225 | 70 | Species-specific | 9 | [36] |
| Jácome et al. 2015 | 2012 | Casanare | *Rhodnius* | *R. prolixus* | 20 | 3 | Species-specific | 8 | [37] |
|  |  |  | *Panstrongylus* | *P. geniculatus* | 2 | 2 |  |  |  |
|  |  |  | *Triatoma* | *T. maculate* | 2 | 1 |  |  |  |
|  |  |  | *Psammolestes* | *P. arthuri* | 2 | 0 |  |  |  |
| Angulo-Silva et al. 2016 | 2008 | Casanare | *Rhodnius* | *R. prolixus* | 56 | 19 | Species-specific | 7 | [17] |
| Hernández et al. 2016 | 2013 | Meta, Guajira, Cesar,Antioquia, Norte de Santander, Casanare y Huila | *Rhodnius* | *R. prolixus* | 77 | 43 | Species-specific | 7 | [38] |
|  |  |  | *Rhodnius* | *R. pictipes* | 8 | 7 |  |  |  |
|  |  |  | *Rhodnius* | *R. pallescens* | 37 | 17 |  |  |  |
|  |  |  | *Panstrongylus* | *P. geniculatus* | 85 | 60 |  |  |  |
|  |  |  | *Triatoma* | *T. maculata* | 34 | 23 |  |  |  |
|  |  |  | *Triatoma* | *T. dimidiata* | 4 | 1 |  |  |  |
| Reyes et al. 2017 | 2014 | Santander | *Rhodnius* | *R. pallescens* | 8 | 4 | Species-specific | 9 | [23] |
|  |  |  | *Panstrongylus* | *P. geniculatus* | 9 | 5 |  |  |  |
| Salazar et al. 2003 | 2002 | Region momposina (Magdalena y bolivar) | *Rhodnius* | *R. pallescens* | 66 | 35 | No species-specific | 9 | [39] |
|  |  |  | *Triatoma* | *T. dimidiate* | 5 | 0 |  |  |  |
|  |  |  | *Eratyrus* | *E. cuspidatus* | 2 | 0 |  |  |  |

**Specie specific:* includes all *T. cruzi* specific methods (culture, PCR and mice inoculation)

* *No species-specific:* includes non-species-specific diagnostic methods (microscopic examination of extruded faeces)

**Table S5. Results of point or pooled prevalence estimates (meta-analysis using random effects model) by detailed diagnostic methods for the different orders of potential *Trypanosoma cruzi* animal reservoirs studied in Colombia.**

| **Order/Family** | **Diagnostic subgroup** | **N° of studies** | **Reservoirs (n)** | **Reservoirs (+)** | **Pooled Prevalence**  **(%)** | **95%CI** | **I^2 (%)^** | **τ^2^** | **p-Value** |
| --- | --- | --- | --- | --- | --- | --- | --- | --- | --- |
|  |  |  |  |  |  |  |  |  |  |
| **Cingulata** | Parasitological | 1 | 21 | 4 | 19 | (5-39) | ---- | ---- | ---- |
| **Pilosa** | Parasitological | 1 | 46 | 0 | 0 | (0-4) | ---- | ---- | ---- |
| **Chiroptera** | Total | 6 | 20267 | 1010 | 15 | (5-29) | 96 | 0.04 | <0.01 |
|  | Parasitological | 2 | 19989 | 936 | 4 | (4-6) | 0 | 0.00 | 0.5 |
|  | Molecular | 3 | 251 | 69 | 23 | (8-44) | 88 | 0.03 | <0.01 |
|  | Mixed | 1 | 27 | 5 | 19 | (6-36) | ---- | ---- | ---- |
| **Rodentia** | Total | 5 | 799 | 59 | 6 | (2-12) | 77 | 0.01 | <0.01 |
|  | Parasitological | 2 | 473 | 40 | 7 | (4-9) | 0 | 0.00 | 0.35 |
|  | Molecular | 3 | 326 | 19 | 8 | (0-21) | 83 | 0.03 | <0.01 |
| **Carnivora** | Total | 11 | 961 | 352 | 19 | (7-35) | 96 | 0.08 | <0.01 |
|  | Serological | 4 | 430 | 196 | 24 | (1-64) | 98 | 0.17 | <0.01 |
|  | Parasitological | 2 | 28 | 0 | 0 | (0-7) | 0 | 0.00 | 0.89 |
|  | Molecular | 4 | 238 | 90 | 17 | (1-42) | 93 | 0.07 | <0.01 |
|  | Mixed | 1 | 242 | 62 | 26 | (2-31) | ---- | ---- | ---- |
| **-          Familly Canidae**  **(domestic dog)** | Total | 10 | 929 | 344 | 19 | (7-36) | 96 | 0.08 | <0.01 |
|  | Serological | 4 | 430 | 196 | 24 | (1-64) | 98 | 0.17 | <0.01 |
|  | Parasitological | 1 | 17 | 0 | 0 | (0-10) | ---- | ---- | ---- |
|  | Molecular | 4 | 238 | 90 | 17 | (1-42) | 93 | 0.07 | <0.01 |
|  | Mixed | 1 | 242 | 62 | 26 | (2-31) | ---- | ---- | ---- |
| **-          Family Felidae (domestic cat)** | Total | 1 | 11 | 0 | 0 | 0-15 | ---- | ---- | ---- |
|  | Parasitological | 1 | 11 | 0 | 0 | (0-15) | ---- | ---- | ---- |
| **Didelphimorphia** | Total | 5 | 310 | 80 | 35 | 16-57 | 92 | 0.06 | <0.01 |
|  | Parasitological | 3 | 173 | 54 | 55 | (9-87) | 96 | 0.19 | <0.01 |
|  | Molecular | 2 | 137 | 26 | 19 | (8-34) | 69 | 0.01 | 0.04 |
| **-*D. marsupialis*** | Total | 5 | 161 | 73 | 48 | 26-71 | 88 | 0.07 | <0.01 |
|  | Parasitological | 3 | 97 | 52 | 63 | (31-90) | 87 | 0.07 | <0.01 |
|  | Molecular | 2 | 64 | 21 | 34 | (5-70) | 88 | 0.86 | <0.01 |
| **- Other Didelphidos** | Total | 3 | 148 | 6 | 3 | 0-10 | 62 | 0.09 | 0.07 |
|  | Parasitological | 1 | 75 | 1 | 1 | (0-6) | ---- | ---- | ---- |
|  | Molecular | 2 | 73 | 5 | 4 | (0-18) | 66 | 0.02 | 0.09 |
| **Primates** | Parasitological | 1 | 343 | 53 | 15 | (12-19) | ---- | ---- | ---- |
| **Other (non-classified)** | Parasitological | 1 | 10 | 2 | 20 | (1-51) | ---- | ---- | ---- |

Serological: methods that include ELISA, Immunofluorescence Tests (IFAT) or Rapid Tests based on whole parasite or recombined antigens.

Mixed: methods, when a combination of the previous methods is conducted

**Table S6. Results of point or pooled prevalence estimates (meta-analysis using random effects model) of *Trypanosoma cruzi* infection by detailed diagnostic methods for the different orders of triatomines studied in Colombia.**

| **Genus/Species** | **Diagnostic method** | **N° of studies** | **Triatomine bugs (n)** | **Triatomine bugs (+)** | **Pooled Prevalence**  **(%)** | **95%CI** | **I^2^**  **^(%)^** | **τ^2^** | **p-Value** |
| --- | --- | --- | --- | --- | --- | --- | --- | --- | --- |
|  |  |  |  |  |  |  |  |  |  |
| ***Belminus herreri*** | **MxMo** | 1 | 44 | 4 | 9.1 | (2-20) | ---- | ---- | ---- |
| ***Belminus ferroae*** | **MxMo** | 1 | 123 | 0 | 0 | 0-1 | ---- | ---- | ---- |
| ***Cavernicola pilosa*** | **NM** | 1 | 28 | 2 | 7 | 0-20 | ---- | ---- | ---- |
| ***Eratyrus cuspidatus*** | **MxMo** | 1 | 16 | 9 | 56 | 31-80 | ---- | ---- | ---- |
| ***Psammolestes arthuri*** | **NM** | 1 | 78 | 0 | 0 | 0-2 | ---- | ---- | ---- |
| ***Pasntrongylus. Rufotuberculatus*** | **Total** | 2 | 160 | 5 | 3 | 0-7 | 22 | 0.001 | 0.26 |
|  | **NM** | 1 | 74 | 1 | 1 | (0-6) | ---- | ---- | ---- |
|  | **MxNM** | 1 | 86 | 4 | 5 | (1-10) | ---- | ---- | ---- |
| ***Panstronglylus. Geniculatus*** | **Total** | 2 | 453 | 70 | 17 | 0-78 | 99 | 0.30 | <0.01 |
|  | **NM** | 1 | 368 | 10 | 1 | (0-3) | ---- | ---- | ---- |
|  | **Mo** | 1 | 85 | 60 | 71 | (6-8) | ---- | ---- | ---- |
| ***Rhodnius prolixus*** | **Total** | 13 | 2023 | 556 | 34 | 17-53 | 99 | 0.13 | <0.01 |
|  | **NM** | 3 | 850 | 71 | 13 | (3-27) | 95 | 0.04 | <0.01 |
|  | **Mo** | 4 | 539 | 364 | 67 | (50-82) | 93 | 0.03 | <0.01 |
|  | **MxMo** | 5 | 561 | 116 | 38 | (11-69) | 97 | 0.11 | <0.01 |
|  | **MxNM** | 1 | 73 | 5 | 7 | (2-14) | ---- | ---- | ---- |
| ***-R. prolixus (domestic)*** | **Total** | 6 | 606 | 105 | 27 | (7-54) | 97 | 0.13 | <0.01 |
|  | **NM** | 3 | 440 | 44 | 19 | (1-51) | 97 | 0.11 | <0.01 |
|  | **Mo** | 1 | 22 | 13 | 59 | (38-79) | NA |  |  |
|  | **MxMo** | 1 | 71 | 43 | 61 | (49-72) | ---- | ---- | ---- |
|  | **MxNM** | 1 | 73 | 5 | 7 | (2-14) | ---- | ---- | ---- |
| ***-R. prolixus (sylvatic)*** | **Total** | 4 | 640 | 334 | 49 | (15-83) | 99 | 0.17 | <0.01 |
|  | **NM** | 1 | 170 | 13 | 8 | (4-12) | ---- | ---- | ---- |
|  | **Mo** | 1 | 429 | 301 | 74 | (47-93) | 97 | 0.04 | <0.01 |
|  | **MxMo** | 2 | 41 | 20 | 47 | (0-10) | 95 | 0.22 | <0.01 |
| ***-R. prolixus (peridomestic)*** | **Total/NM** | 1 | 10 | 0 | 0 | 0-17 | ---- | ---- | ---- |
| ***-R. prolixus (not specified)*** | **Total** | 4 | 746 | 107 | 22 | (6-45) | 97 | 0.06 | <0.01 |
|  | **NM** | 1 | 220 | 11 | 5 | (2-8) | ---- | ---- | ---- |
|  | **Mo** | 1 | 77 | 43 | 56 | (45-67) | ---- | ---- | ---- |
|  | **MxMo** | 2 | 449 | 53 | 19 | (1-49) | 95 | 0.05 | <0.01 |
| ***Rhodnius. Pallescens*** | **Total** | 7 | 383 | 227 | 54 | 40-68 | 84 | 0.02 | <0.01 |
|  | **NM** | 1 | 66 | 35 | 53 | (41-65) | ---- | ---- | ---- |
|  | **Mo** | 2 | 103 | 56 | 54 | (41-66) | 38 | 0 | 0.2 |
|  | **MxMo** | 3 | 203 | 136 | 70 | (58-81) | 50 | 0 | 0.13 |
|  | **MxNM** | 1 | 11 | 0 | 0 | (0-15) | ---- | ---- | ---- |
| ***Rhodnius pictipes*** | **Total** | 2 | 21 | 1 | 3 | 0-18 | 0 | 0 | 0.34 |
|  | **NM** | 1 | 11 | 1 | 9 | (0-5) | ---- | ---- | ---- |
|  | **MxMo** | 1 | 10 | 0 | 0 | (0-17) | ---- | ---- | ---- |
| ***Triatoma maculate*** | **Total** | 7 | 342 | 161 | 33 | (8-65) | 97 | 0.19 | <0.01 |
|  | **NM** | 3 | 129 | 16 | 6 | (0-38) | 94 | 0.14 | <0.01 |
|  | **Mo** | 1 | 34 | 23 | 68 | (51-82) | ---- | ---- | ---- |
|  | **MxMo** | 3 | 179 | 122 | 68 | (61-75) | 0 | 0 | 0.4 |
| ***Triatoma dimidiate*** | **Total** | 4 | 3063 | 314 | 21 | (7-40) | 98 | 0.05 | <0.01 |
|  | **NM** | 1 | 2671 | 222 | 25 | (0-74) | ---- | ---- | ---- |
|  | **MxMo** | 2 | 281 | 80 | 25 | (14-39) | 75 | 0.01 | 0.05 |
|  | **MxNM** | 1 | 111 | 12 | 11 | (6-17) | ---- | ---- | ---- |
| ***Triatoma venosa*** | **NM** | 1 | 1115 | 5 | 0.004 | 0-1 | ---- | ---- | ---- |
| **Non-classified** | **Total** | 4 | 3293 | 320 | 17 | (7-30) | 89 | 0.03 | <0.01 |

NM: non-molecular methods

Mo: molecular methods

MxMo microscopic examination of extruded faeces and PCR

MxNM: microscopic examination of extruded faeces and mice inoculation

**Table S7. Results of Eggerʼs test for publication bias assessment for estimates of *Trypanosoma cruzi* infection rates in animal reservoirs and triatomine vectors in Colombia.**

|  | **Taxonomic classification** | **Diagnostic subgroup** | **N° of studies** | **Pooled Prevalence(%)** | **95%CI** | **p-value Egger test** |
| --- | --- | --- | --- | --- | --- | --- |
| **Potential Reservoirs** | ***- Family Canidae*** | **Serological** | 4 | 24 | (1-64) | ---- |
|  |  | **Non-serological** | 5 | 14 | (1-37) | 0.013 |
|  |  | **Mixed** | 1 | 26 | (2-31) | ---- |
|  | ***-  Family Felidae*** | **Non-serological** | 1 | 0 | (0-15) | ---- |
|  | **Chiroptera** | **Non-serological** | 6 | 15 | (5-29) | 0.10 |
|  | **Didelphimorphia** | **Non-serological** | 5 | 35 | (16-57) | 0.16 |
|  | **-*D. marsupialis*** | **Non-serological** | 5 | 48 | (26-71) | 0.60 |
|  | **- Other Didelphidos** | **Non-serological** | 3 | 3 | (0-10) | ---- |
|  | **Rodentia** | **Non-serological** | 5 | 6 | (2-12) | 0.86 |
| **Triatomines** | ***R. pallescens*** | ***T.cruzi*** | 6 | 54 | (37-71) | 0.91 |
|  |  | ***T.spp*** | 1 | 53 | (41-65) | ---- |
|  | ***R. prolixus*** | ***T.cruzi*** | 10 | 46 | (23-69) | 0.50 |
|  |  | ***T.spp*** | 3 | 13 | (3-27) | ---- |
|  | ***-R. prolixus (domestic)*** | ***T.cruzi*** | 3 | 39 | (4-82) | ---- |
|  |  | ***T.spp*** | 3 | 19 | (1-51) | ---- |
|  | ***-R. prolixus (sylvatic)*** | ***T.cruzi*** | 4 | 62 | (38-84) | ---- |
|  |  | ***T.spp*** | 1 | 8 | (4-12) | ---- |
|  | ***-R. prolixus (peridomestic)*** | ***T.spp*** | 1 | 0 | (0-17) | ---- |
|  | ***-R. prolixus (not specified)*** | ***T.cruzi*** | 3 | 30 | (5-66) | ---- |
|  |  | ***T.spp*** | 1 | 5 | (2-8) | ---- |
|  | ***T. dimidiate*** | ***T.cruzi*** | 3 | 20 | (8-35) | 0.48 |
|  |  | ***T.spp*** | 1 | 25 | (0-74) | ---- |
|  | ***T. maculate*** | ***T.cruzi*** | 4 | 68 | (62-74) | 0.12 |
|  |  | ***T.spp*** | 3 | 6 | (0-38) | ---- |

**Table S8. Results of point or pooled prevalence estimates (meta-analysis using random effects model) for the different orders of potential *Trypanosoma cruzi* reservoirs studied in Colombia, using only high-quality reports.**

| **Order/Family** | **Diagnostic subgroup** | **N° of studies** | **Reservoirs (n)** | **Reservoirs (+)** | **Pooled Prevalence**  **(%)** | **95%CI** | **I^2^**  **^(%)^** | **τ^2^** | **p-Value** |
| --- | --- | --- | --- | --- | --- | --- | --- | --- | --- |
|  |  |  |  |  |  |  |  |  |  |
| **Carnivora** | **Non-serological** | 6 | 638 | 252 | 20 | (3-47) | 98 | 0.11 | <0.01 |
| **- Family Canidae** | **Serological** | 1 | 60 | 3 | 5 | (1-12) | ---- | ---- | ---- |
|  | **Non-serological** | 2 | 254 | 174 | 30 | (0-100) | 97 | 0.35 | <0.01 |
|  | **Mixed** | 2 | 308 | 72 | 21 | (12-32) | 69 | 0.01 | 0.07 |
| **-  Family Felidae** | **Non-serological** | 1 | 11 | 0 | 0 | (0-15) | ---- | ---- | ---- |
| **Chiroptera** | **Non-serological** | 3 | 20016 | 941 | 6 | (2-12) | 71 | 0.00 | 0.03 |
| **Didelphimorphia** | **Non-serological** | 2 | 150 | 34 | 37 | (3-79) | 89 | 0.09 | <0.01 |
| **-*D. marsupialis*** | **Non-serological** | 2 | 75 | 33 | 47 | (28-66) | 46 | 0.01 | 0.17 |
| **- Other didelphis** | **Non-serological** | 1 | 75 | 1 | 1 | (0-6) | ---- | ---- | ---- |
| **Rodentia** | **Non-serological** | 2 | 473 | 40 | 7 | (4-9) | 0 | 0.00 | 0.35 |

Non-serological: includes parasitological methods (blood smears hemoculture and xenodiagnostic) and molecular methods (PCR).

Serological: includes ELISA, Immunofluorescence Tests (IFAT) or Rapid Tests based on whole parasite or recombined antigens.

**Table S9. Results of point or pooled prevalence estimates (meta-analysis using random effects model) of *Trypanosoma cruzi* infection for the different orders of triatomines studied in Colombia, using only high-quality reports.**

| **Genus/Species** | | **N° of studies** | **Triatomine bugs (n)** | **Triatomine bugs (+)** | **Pooled Prevalence (%)** | **95%CI** | **I^2^**  **^(%)^** | **τ^2^** | **p-Value** |
| --- | --- | --- | --- | --- | --- | --- | --- | --- | --- |
|  |  |  |  |  |  |  |  |  |  |
| ***Belminus ferroae*** | ***T.cruzi*** | 1 | 123 | 0 | 0 | (0-1) | ---- | ---- | ---- |
| ***Belminus herreri*** | ***T.cruzi*** | 1 | 44 | 4 | 0 | (2-20) | ---- | ---- | ---- |
| ***Cavernicola pilosa*** | ***T.spp*** | 1 | 28 | 2 | 7 | (0-20) | ---- | ---- | ---- |
| ***Psammolestes arthuri*** | ***T.spp*** | 1 | 78 | 0 | 0 | (0-2) | ---- | ---- | ---- |
| ***P. rufotuberculatus*** | ***T.cruzi*** | 1 | 86 | 4 | 5 | (1-10) | ---- | ---- | ---- |
| ***R. pallescens*** | ***T.cruzi*** | 3 | 135 | 83 | 43 | (11-79) | 94 | 0.09 | <0.01 |
|  | ***T.spp*** | 1 | 66 | 35 | 53 | (41-65) | ---- | ---- | ---- |
| ***R. pictipes*** | ***T.spp*** | 1 | 11 | 1 | 9 | (0-35) | ---- | ---- | ---- |
| ***R. prolixus*** | ***T.cruzi*** | 6 | 378 | 227 | 51 | (19-83) | 97 | 0.17 | <0.01 |
|  | ***T.spp*** | 2 | 284 | 48 | 17 | (12-21) | 0 | 0.00 | 0.57 |
| ***-R. prolixus (domestic)*** | ***T.cruzi*** | 3 | 166 | 61 | 39 | (4-82) | 97 | 0.15 | <0,01 |
|  | ***T.spp*** | 2 | 114 | 34 | 29 | (16-43) | 53 | 0.01 | 0.14 |
| ***-R. prolixus (sylvatic)*** | ***T.cruzi*** | 3 | 210 | 164 | 62 | (20-96) | 95 | 0.14 | <0,01 |
|  | ***T.spp*** | 1 | 170 | 13 | 8 | (4-12) | ---- | ---- | ---- |
| ***-R. prolixus (peridomestic)*** | ***T.spp*** | 1 | 10 | 0 | 0 | (0-17) | ---- | ---- | ---- |
| ***-R. prolixus (not specified)*** | ***T.cruzi*** | 1 | 56 | 19 | 34 | (22-47) | ---- | ---- | ---- |
| ***T. dimidiate*** | ***T.cruzi*** | 2 | 336 | 82 | 20 | (5-43) | 95 | 0.03 | <0.01 |
| ***T. maculate*** | ***T.cruzi*** | 3 | 179 | 122 | 68 | (61-75) | 0 | 0 | 0.4 |
|  | ***T.spp*** | 2 | 42 | 16 | 21 | (0-89) | 95 | 0.25 | <0.01 |
| **Non-classified vectors** | ***T.cruzi*** | 2 | 3145 | 290 | 16 | (3-36) | 89 | 0.03 | <0.01 |
|  | ***T.spp*** | 1 | 40 | 0 | 0 | (0-4) | ---- | ---- | ---- |

*T. cruzi:* includes all *T.cruzi* specific methods (culture, PCR and mice inoculation)

*T. spp:* includes non-species-specific diagnostic methods (direct observation based on microscopic examination of extruded faeces)

**Figure S1. Content quality assessment of the 39 studies used in the meta-analysis.**


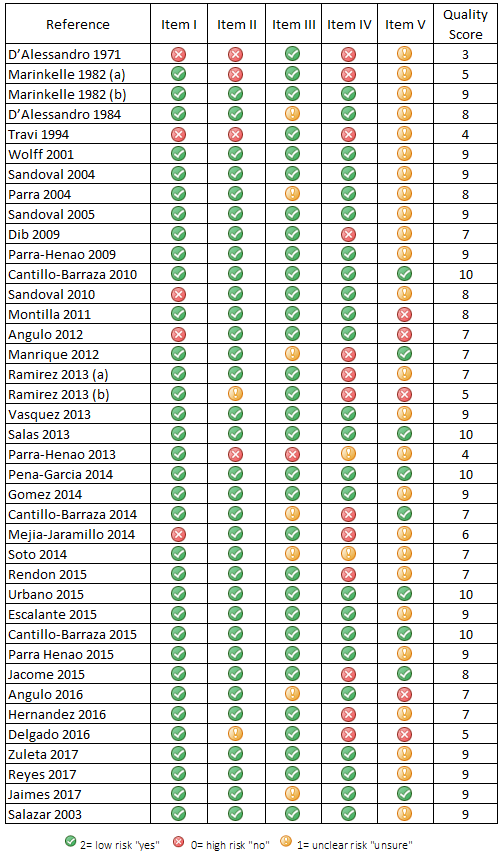


**Figure S2.** **Funnel plot of the 95%CI pseudo limits for the estimates of *Trypanosoma cruzi* prevalence in animal reservoirs in Colombia**

**
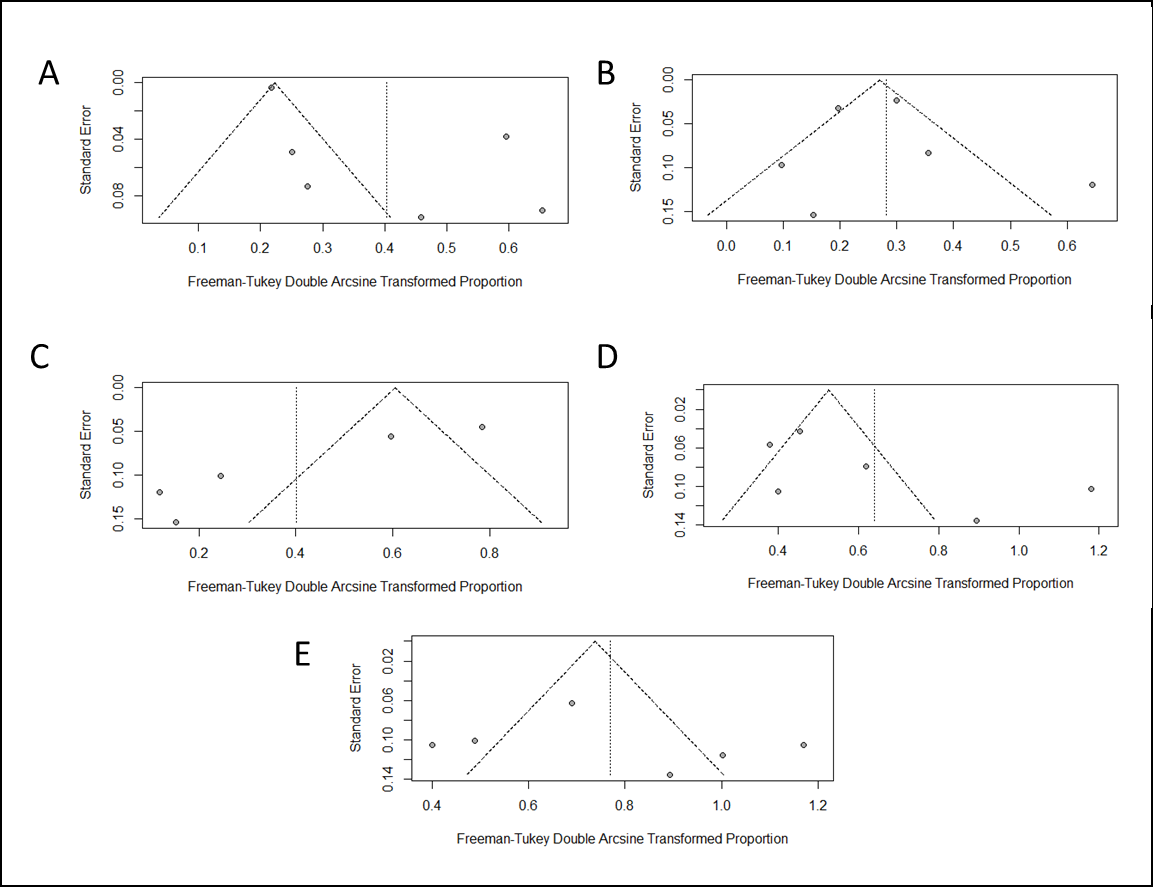
**

Order Chiroptera (Panel A), Order Rodentia (Panel B), Family Canidae diagnostic subgroup non-serological methods (Panel C), Order Didelphimorphia (Panel D), *Didelphis masupialis* (Panel E). On the right axis of the log (prevalence) from each study is plotted against its corresponding standard error). Lines represented pseudo 95% confidence limits.

**Figure S3. Funnel plot of the 95%CI pseudo limits for the estimates of *Trypanosoma cruzi* prevalence in family Canidae in Colombia**

On the right axis of the log (prevalence) from each study is plotted against its corresponding standard error. Filled circles represented the studies included in the meta-analysis and the empty circles represent the hypothetically missing studies. Lines represented pseudo 95% confidence limits for the re-estimate using the 'filled' studies

**
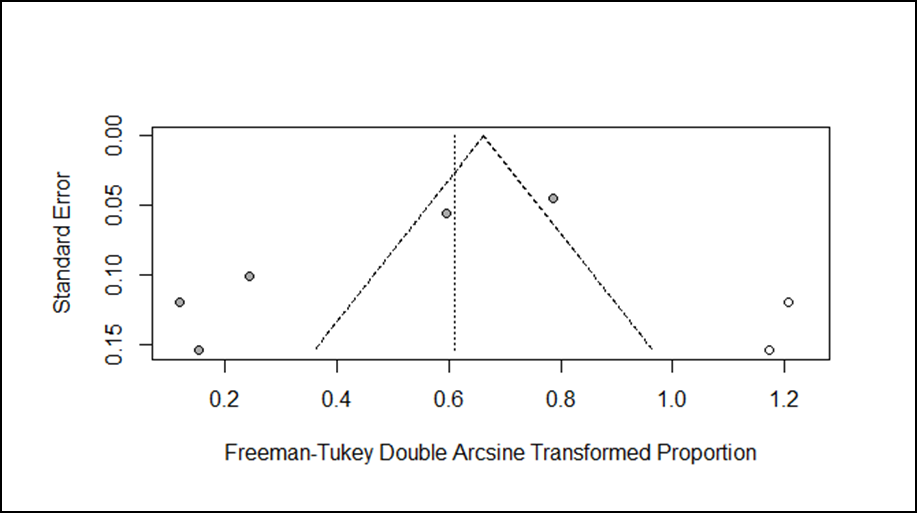
**

**Figure S4. Funnel plot of the 95%CI pseudo limits for the estimates of *Trypanosoma cruzi* prevalence in triatomine species in Colombia**

On the right axis of the log (prevalence) from each study is plotted against its corresponding standard error. Lines represented pseudo 95% confidence limits*.*

**
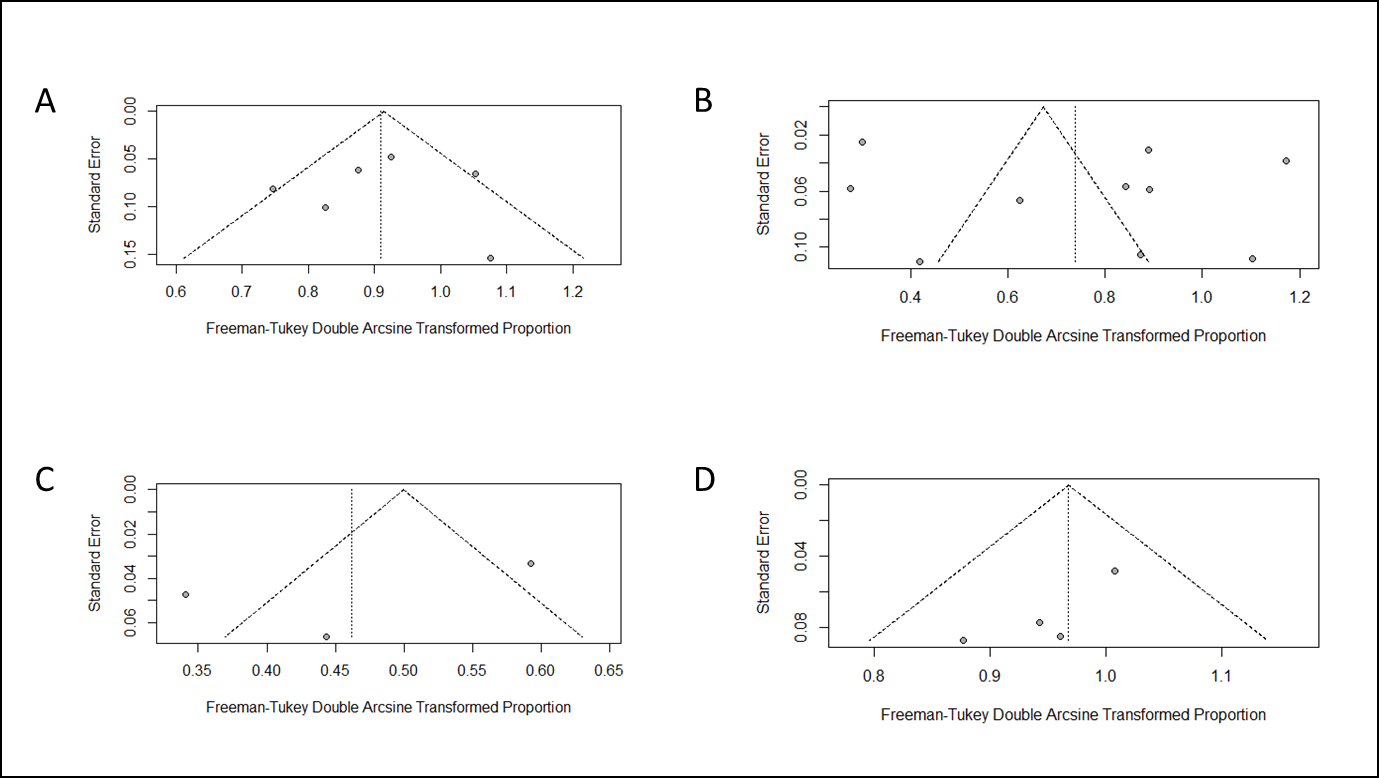
**

*Rhodnius pallescens* (Panel A), *Rhodnius prolixus* (Panel B), *Triatoma dimidiate* (Panel C), *Triatoma maculata* (Panel D).

**Figure S5. Sensitivity analyses for estimates of the prevalence of *Trypanosoma cruzi* in potential animal reservoirs in Colombia, by omitting one study at a time.**

The figure shows the forest plot re-estimated effect after removing one single study

**
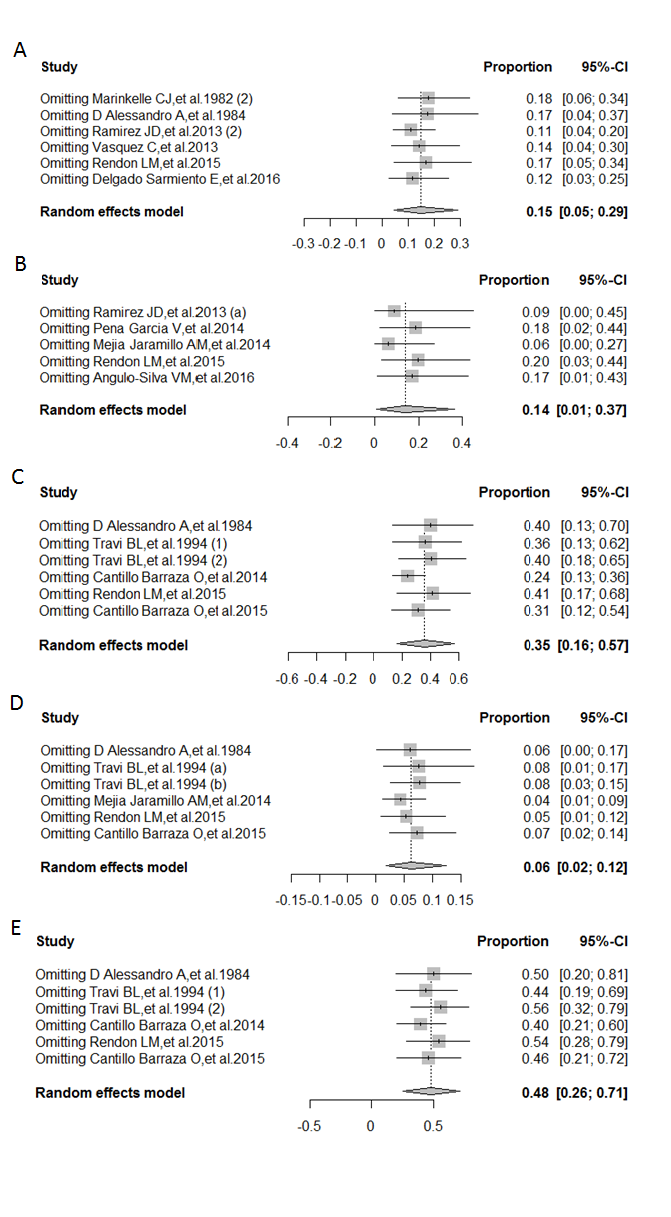
**

Order Chiroptera (Panel A), Order Rodentia (Panel B), Family *Canidae* diagnostic subgroup no serological (Panel C), Order Didelphimorphia (Panel D), *Didelphis masupialis* (Panel E).

**Figure S6. Sensitivity analyses for estimates of the prevalence of *Trypanosoma cruzi* in triatomine vectors in Colombia, by omitting one study at a time.**

The figure shows the forest plot re-estimated effect after removing any single study

**
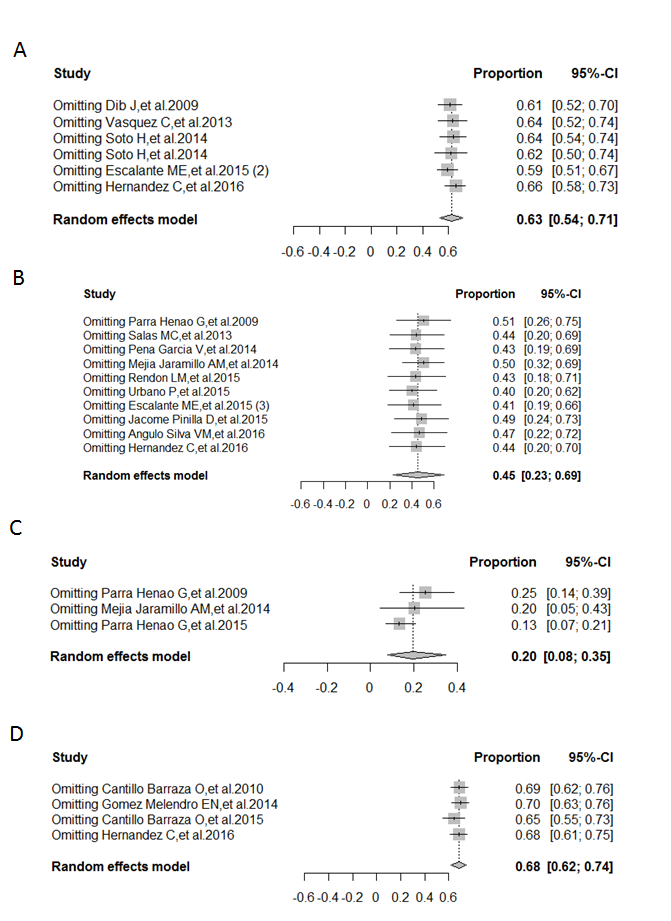
**

*Rhodnius pallescens* (Panel A), *Rhodnius prolixus* (Panel B), *Triatoma dimidiate* (Panel C), *Triatoma maculata* (Panel D).

**Figure S7. Distribution of the quality assessment scores among the 39 studies included in the meta-analysis.**

**
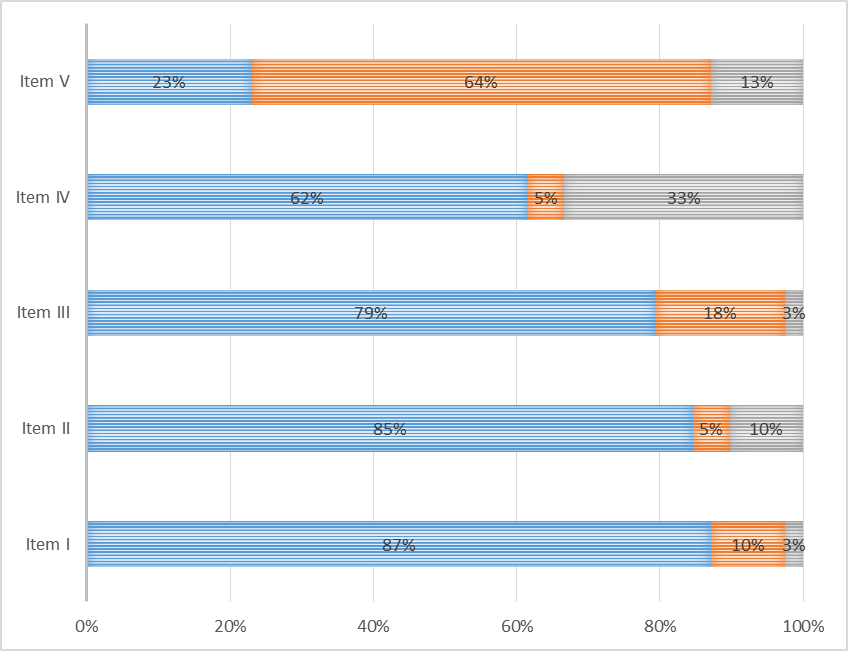
**

- **Low risk of bias**
- **Unclear risk "unsure"**
- **High risk of bias**

**References**

1. Ding H, Gao Y-M, Deng Y, Lamberton PHL, Lu D-B. A systematic review and meta-analysis of the seroprevalence of *Toxoplasma gondii* in cats in mainland China. Parasit Vectors. BioMed Central; 2017;10:27.

2. D’Alessandro A, Wells EA. Trypanosome infections in the family Cervidae. Trans R Soc Trop Med Hyg. 1971;65:845.

3. Marinkelle CJ. The prevalence of *Trypanosoma cruzi* infection in Colombia monkey and marmosets. Ann Trop Med Parasitol. 1982;76:121–4.

4. Marinkelle CJ. Prevalence of *Trypanosoma cruzi*-like infection of Colombian bats. Ann Trop Med Parasitol. 1982;76:125–34.

5. D’Alessandro A, Barreto P, Saravia N, Barreto M. Epidemiology of *Trypanosoma cruzi* in the oriental plains of Colombia. Am J Trop Med Hyg. 1984;33:1084–95.

6. Travi BL, Jaramillo C, Montoya J, Segura I, Zea A, Goncalves A, et al. *Didelphis marsupialis*, an important reservoir of *Trypanosoma* (Schizotrypanum) *cruzi* and *Leishmania* (Leishmania) *chagasi* in Colombia. Am J Trop Med Hyg. 1994;50:557–65.

7. Wolff M, Castillo D, Uribe J, Arboleda JJ. American trypanosomiasis: determination of epidemiologic transmission risk in Amalfi, Antioquia, Colombia. Iatreia. 2001;14:111–21.

8. Manrique-Abril D, Manrique-Abril F, Lorca MH, Ospina JD. Prevalencia de anticuerpos para *Trypanosoma cruzi* en caninos de dos municipios endémicos de Boyacá Prevalence of antibodies for *Trypanosoma cruzi* in canines from two endemic municipalities of Boyacá. 2012;17.

9. Ramírez JD, Turriago B, Tapia-Calle G, Guhl F. Understanding the role of dogs (Canis lupus familiaris) in the transmission dynamics of *Trypanosoma cruzi* genotypes in Colombia. Vet Parasitol. 2013;196:216–9.

10. Ramírez JD, Tapia-Calle G, Muñoz-Cruz G, Poveda C, Rendón LM, Hincapié E, et al. Trypanosome species in neo-tropical bats: biological, evolutionary and epidemiological implications. Infect Genet Evol. 2014;22:250–6.

11. Vásquez C, Robledo S, Calle J, Triana O. [Identification of new epidemiological scenarios for Chagas disease in the Momposina region, North of Colombia]. Biomedica. Instituto Nacional de Salud; 2013;33:526–37.

12. Peña-García VH, Gómez-Palacio AM, Triana-Chávez O, Mejía-Jaramillo AM. Eco-Epidemiology of Chagas Disease in an Endemic Area of Colombia: Risk Factor Estimation, *Trypanosoma cruzi* Characterization and Identification of Blood-Meal Sources in Bugs. Am J Trop Med Hyg. 2014;91:1116–24.

13. Cantillo-Barraza O, Chaverra D, Marcet P, Arboleda-Sánchez S, Triana-Chávez O. *Trypanosoma cruzi* transmission in a Colombian Caribbean region suggests that secondary vectors play an important epidemiological role. Parasit Vectors. 2014;7:381.

14. Mejía-Jaramillo AM, Agudelo-Uribe LA, Dib JC, Ortiz S, Solari A, Triana-Chávez O. Genotyping of *Trypanosoma cruzi* in a hyper-endemic area of Colombia reveals an overlap among domestic and sylvatic cycles of Chagas disease. Parasit Vectors. 2014;7:108.

15. Rendón LM, Guhl F, Cordovez JM, Erazo D. New scenarios of *Trypanosoma cruzi* transmission in the Orinoco region of Colombia. Mem Inst Oswaldo Cruz. 2015;110:283–8.

16. Cantillo-Barraza O, Garcés E, Gómez-Palacio A, Cortés LA, Pereira A, Marcet PL, et al. Eco-epidemiological study of an endemic Chagas disease region in northern Colombia reveals the importance of *Triatoma maculata* (Hemiptera: Reduviidae), dogs and *Didelphis marsupialis* in *Trypanosoma cruzi* maintenance. Parasit Vectors. 2015;8:482.

17. Angulo-Silva VM, Castellanos-Domínguez YZ, Flórez-Martínez M, Esteban-Adarme L, Pérez-Mancipe W, Farfán-García AE, et al. Human trypanosomiasis in the eastern plains of Colombia: New transmission scenario. Am J Trop Med Hyg. 2016;94:348–51.

18. Delgado-Sarmiento E, Herrera-Sepúlveda M-T, Pavía P, Pérez-Torres J, Cuervo CL. *Trypanosoma cruzi* infection in the heart of Colombian wild bats. Int J Infect Dis. 2016;45:356–7.

19. Zuleta-Dueñas LP, López-Quiroga ÁJ, Torres-Torres F, Castañeda-Porras O. Possible oral transmission of Chagas disease among hydrocarbons sector workers in Casanare, Colombia, 2014. Biomedica. 2017;37:218–32.

20. Jaimes-Dueñez J, Triana-Chávez O, Cantillo-Barraza O, Hernández C, Ramírez JD, Góngora-Orjuela A. Molecular and serological detection of *Trypanosoma cruzi* in dogs (*Canis lupus familiaris*) suggests potential transmission risk in areas of recent acute Chagas disease outbreaks in Colombia. Prev Vet Med. 2017;141:1–6.

21. Parra GJ, Restrepo Isaza M, Restrepo BN, Domínguez J de D. Estudio de tripanosomiasis americana en dos poblados indígenas de la Sierra Nevada de Santa Marta, Colombia. CES Med. 2004;18:43–50.

22. Soto H, Tibaduiza T, Montilla M, Triana O, Suárez DC, Torres MT, et al. Investigación de vectores y reservorios en brote de Chagas agudo por posible transmisión oral en Aguachica, Cesar, Colombia. Cad Saude Publica. 2014;30:746–56.

23. Reyes M, Torres Á, Lyda E, Flórez M, Angulo V. Riesgo de transmisión de la enfermedad de Chagas por intrusión de triatominos y mamíferos silvestres en la ciudad de Bucaramanga (Santander, Colombia). Biomédica Rev del Inst Nac Salud. 2013;33:24.

24. Sandoval CM, Duarte R, Gutíerrez R, Rocha D da S, Angulo VM, Esteban L, et al. Feeding sources and natural infection of Belminus herreri (Hemiptera, Reduviidae, Triatominae) from dwellings in Cesar, Colombia. Mem Inst Oswaldo Cruz. 2004;99:137–40.

25. Dib J, Barnabe C, Tibayrenc M, Triana O. Incrimination of *Eratyrus cuspidatus* (Stal) in the transmission of Chagas’ disease by molecular epidemiology analysis of *Trypanosoma cruzi* isolates from a geographically restricted area in the north of Colombia. Acta Trop. 2009;111:237–42.

26. Parra-Henao G, Angulo V, Jaramillo N, Restrepo M. Triatominos (Hemiptera: Reduviidae) de la Sierra Nevada de Santa Marta, Colombia. Aspectos epidemiológicos, entomológicos y de distribución. Rev CES Med. 2009;23:17–26.

27. Cantillo-Barraza O, Gómez-Palacio A, Salazar D, Mejía-Jaramillo AM, Calle J, Triana O. Distribution and ecoepidemiology of the triatomine fauna (Hemiptera: Reduviidae) in Margarita Island, Bolívar, Colombia. Biomedica. 2010;30:382–9.

28. Sandoval CM, Ortiz N, Jaimes D, Lorosa E, Galvão C, Rodriguez O, et al. Feeding behaviour of Belminus ferroae (Hemiptera: Reduviidae), a predaceous Triatominae colonizing rural houses in Norte de Santander, Colombia. Med Vet Entomol. 2010;24:124–31.

29. Montilla M, Soto H, Parra E, Torres M, Carrillo P, Lugo L, et al. Infestation by triatomine bugs in indigenous communities of Valledupar, Colombia. Rev Saude Publica. 2011;45:773–80.

30. Angulo VM, Esteban L, Luna KP. Attalea butyracea palms adjacent to housing as a source of infestation by Rhodnius prolixus (Hemiptera: Reduviidae). Biomedica. 2012;32:277–85.

31. Castro-Salas M. Aspectos ecoepidemiológicos en la transmisión de la enfermedad de chagas en Santa Rosalia , Vichada Aspectos ecoepidemiológicos en la transmisión de la enfermedad de chagas en Santa Rosalía , Vichada. 2013;1–101.

32. Parra-Henao GJ, Florez M, Ángulo VM, Red Chagas Colombia, editores. Vigilancia de Triatominae (Hemiptera: Reduviridae) en Colombia. In: Memorias del Curso de Capacitación Métodos Básicos en Epidemiología y Redacción Científica. Bogotá: Sic Editorial; 2015.

33. Gómez-Melendro EN, Hernández C, González-Uribe C, Brochero H. First Record of *Triatoma maculata* (Erichson, 1848) (Hemiptera: Reduviidae: Triatomini) in the Municipality of Riohacha, La Guajira - Colombia. Front public Heal. 2014;2:219.

34. Urbano P, Poveda C, Molina J. Effect of the physiognomy of *Attalea butyracea* (Arecoideae) on population density and age distribution of *Rhodnius prolixus* (Triatominae). Parasit Vectors. 2015;8:199.

35. Escalante ME, Gomez D, Silvera LA, Sánchez G, Venegas J. Detection of high percentage of *Trypanosoma cruzi* infection, the etiologic agent of Chagas disease, in wild populations of Colombian Caribbean triatomines. Acta Parasitol. 2015;60:315–21.

36. Parra-Henao G, Angulo VM, Osorio L, Jaramillo-O N. Geographic Distribution and Ecology of *Triatoma dimidiata* (Hemiptera: Reduviidae) in Colombia. J Med Entomol. 2016;53:122–9.

37. Jácome-Pinilla D, Hincapie-Peñaloza E, Ortiz MI, Ramírez JD, Guhl F, Molina J. Risks associated with dispersive nocturnal flights of sylvatic Triatominae to artificial lights in a model house in the northeastern plains of Colombia. Parasit Vectors. 2015;8:600.

38. Hernández C, Salazar C, Brochero H, Teherán A, Buitrago LS, Vera M, et al. Untangling the transmission dynamics of primary and secondary vectors of *Trypanosoma cruzi* in Colombia: Parasite infection, feeding sources and discrete typing units. Parasites and Vectors [Internet]. 2016 [cited 2017 Oct 2];9:620.

39. Salazar DA, Calle J. Caracterización ecoepidemiológica de *Rhodnius pallescens* en la palma *Attalea butyracea* en la región Momposina (Colombia). Actual Biológicas Univ Antioquia. Veterinaria Organización; 2003;25:31–8.
